# Feasibility Study of EndoTAG-1, a Tumor Endothelial Targeting Agent, in Combination with Paclitaxel followed by FEC as Induction Therapy in HER2-Negative Breast Cancer

**DOI:** 10.1371/journal.pone.0154009

**Published:** 2016-07-25

**Authors:** Michail Ignatiadis, Dimitrios Zardavas, Marc Lemort, Celine Wilke, Marie-Catherine Vanderbeeken, Veronique D’Hondt, Evandro De Azambuja, Andrea Gombos, Fabienne Lebrun, Lissandra Dal Lago, Fanny Bustin, Marion Maetens, Lieveke Ameye, Isabelle Veys, Stefan Michiels, Marianne Paesmans, Denis Larsimont, Christos Sotiriou, Jean-Marie Nogaret, Martine Piccart, Ahmad Awada

**Affiliations:** 1 Medical Oncology Clinic, Institut Jules Bordet, Université Libre de Bruxelles, Brussels, Belgium; 2 Breast Cancer Translational Research Laboratory, Institut Jules Bordet, Université Libre de Bruxelles, Brussels, Belgium; 3 Breast International Group (BIG aisbl), Brussels, Belgium; 4 Department of Radiology, Institut Jules Bordet, Université Libre de Bruxelles, Brussels, Belgium; 5 Medigene AG, Planegg, Germany; 6 Department of Biostatistics, Institut Jules Bordet, Université Libre de Bruxelles, Brussels, Belgium; 7 Department of Surgery, Institut Jules Bordet, Université Libre de Bruxelles, Brussels, Belgium; 8 Service de Biostatistique et d’Epidémiologie, Gustave Roussy, Univ. Paris-Sud, Villejuif, France; 9 Department of Pathology, Institut Jules Bordet, Université Libre de Bruxelles, Brussels, Belgium; Ohio State University, UNITED STATES

## Abstract

**Background:**

EndoTAG-1, a tumor endothelial targeting agent has shown activity in metastatic triple-negative breast cancer (BC) in combination with paclitaxel.

**Methods:**

HER2-negative BC patients candidates for neoadjuvant chemotherapy were scheduled to receive 12 cycles of weekly EndoTAG-1 22mg/m^2^ plus paclitaxel 70mg/m^2^ followed by 3 cycles of FEC (Fluorouracil 500mg/m^2^, Epirubicin 100mg/m^2^, Cyclophosphamide 500mg/m^2^) every 3 weeks followed by surgery. Primary endpoint was percent (%) reduction in Magnetic Resonance Imaging (MRI) estimated Gadolinium (Gd) enhancing tumor volume at the end of EndoTAG-1 plus paclitaxel administration as compared to baseline. Safety, pathological complete response (pCR) defined as no residual tumor in breast and axillary nodes at surgery and correlation between % reduction in MRI estimated tumor volume and pCR were also evaluated.

**Results:**

Fifteen out of 20 scheduled patients were included: Six patients with estrogen receptor (ER)-negative/HER2-negative and 9 with ER-positive/HER2-negative BC. Nine patients completed treatment as per protocol. Despite premedication and slow infusion rates, grade 3 hypersensitivity reactions to EndoTAG-1 were observed during the 1^st^, 2^nd^, 3^rd^ and 6^th^ weekly infusion in 4 patients, respectively, and required permanent discontinuation of the EndoTAG-1. Moreover, two additional patients stopped EndoTAG-1 plus paclitaxel after 8 and 9 weeks due to clinical disease progression. Two patients had grade 3 increases in transaminases and 1 patient grade 4 neutropenia. pCR was achieved in 5 of the 6 ER-/HER2- and in none of the 9 ER+/HER2- BC patients. The mean % reduction in MRI estimated tumor volume at the end of EndoTAG-1 plus paclitaxel treatment was 81% (95% CI, 66% to 96%, p<0.001) for the 15 patients that underwent surgery; 96% for patients with pCR and 73% for patients with no pCR (p = 0.04).

**Conclusions:**

The EndoTAG-1 and paclitaxel combination showed promising preliminary activity as preoperative treatment, especially in ER-/HER2- patients. Further studies are warranted with need of premedication optimization.

**Trial Registration:**

ClinicalTrials.gov NCT01537536

## Introduction

Breast cancer (BC) represents the most frequently diagnosed malignancy and the leading cause of cancer death among women in the western world [1]. Patients diagnosed with early stage disease have high survival rates with the local and systemic treatment options available. Neoadjuvant chemotherapy (NAC) was introduced as a way to improve surgical options for BC, being administered more commonly to patients with high risk factors such as larger tumors and/or lymph node involvement at diagnosis [2]. NAC has the potential to reduce tumor burden, thus achieving down-staging of the disease allowing breast-conserving surgery (BCS), treats micrometastatic tumor burden, and allows for *in vivo* assessment of tumor response to therapy [3]. Although no specific NAC regimen is considered as the gold standard, anthracycline-and taxane-based chemotherapy is often used, especially for patients with high-risk, node-positive disease [4].

Pathological complete response (pCR) after NAC has been associated with significantly longer recurrence free (RFS) and overall survival (OS) [5–9]. Moreover, in the case of triple negative breast cancer (TNBC), representing an aggressive BC subtype with poor clinical outcome, patients achieving pCR after NAC have excellent prognosis [10].

In terms of radiologic monitoring of response to NAC, dynamic contrast-enhanced magnetic resonance imaging (MRI) can discriminate non-vascularized NAC-induced fibrosis from residual vital tumor burden [11,12]. Moreover, MRI assessment during NAC can allow early identification of non-responders. Recent data supports that MRI performed during NAC provides an accurate way to monitor response in TNBC or HER2-positive disease [13]. Thus, MRI holds the potential to guide the NAC strategy, through in vivo monitoring of response to the treatment provided.

EndoTAG-1 is a cationic liposomal paclitaxel that has shown activity in TNBC when used in combination with paclitaxel. In a randomized controlled phase II study, TNBC patients with 0–1 prior chemotherapy regimens for metastatic disease and a (neo)-adjuvant taxane free interval of > 6 months, the clinical benefit rate (complete or partial response at any time and stable disease ≥ 6 months) on combination of EndoTAG-1 and paclitaxel (n = 50 patients) was 53% compared to 31% and 36% on EndoTAG-1 and paclitaxel monotherapy, respectively [14]. EndoTAG-1 showed a similar safety profile to paclitaxel. On combination treatment, a slight increase in grade 3/4 adverse events (AEs) was observed compared to either monotherapy, with neutropenia being the most frequent AE.

In the study reported here, we investigated the combination of EndoTAG-1/Paclitaxel followed by Fluorouracil, Epirubicin, Cyclophosphamide (FEC) as NAC in HER2-negative BC. The trial is registered at clinicaltrials.gov (NCT01537536).

## Patients and Methods

### Eligibility

This study was conducted at the Institute Jules Bordet (IJB), Brussels, Belgium. Patients were eligible if they had newly diagnosed histologically confirmed BC, candidate for NAC. Histology grade > 1 and HER2-negativity as assessed by either immunohistochemistry (IHC) or fluorescent in-situ hybridization (FISH) were also required. Additional inclusion criteria were female gender; age ≥18 years old; ECOG performance status ≤1; willingness to perform double-barrier-contraception during study and for 6 months post chemotherapy treatment; adequate organ function. Patients with pregnancy or nursing status, known positive HIV testing, known hypersensitivity to any component of the EndoTAG-1, taxanes or FEC formulations and history of malignancy other than breast cancer <5 years prior to enrollment were ineligible to this study.

### Study Design

This was a prospective, single-center, open-label phase II clinical study investigating the feasibility and antitumor activity of EndoTAG-1/Paclitaxel combination therapy in patients with HER2 negative BC candidate for NAC, as measured by the decrease in MRI-estimated tumor volume at the end of EndoTAG-1/Paclitaxel administration. The TREND checklist (S1 Fig) and the full study protocol (S1 Text) are provided as supplementary information.

Primary endpoint of the study was the percent reduction in MRI-estimated Gd-enhancing tumor volume at the end of EndoTAG-1/Paclitaxel treatment compared to baseline. Secondary endpoints included the percent reduction in linear tumor size as measured on MRI; the percent reduction in MRI- measured transfer constant Ktrans (evaluating quality of tumour perfusion) at the end of EndoTAG-1/Paclitaxel administration (Ktrans was obtained using the pharmacokinetic modelling as described by. Tofts PS et al [15]); pathological complete response (pCR) rate, defined as the absence of any residual invasive cancer in the breast and the absence of any metastatic cells in the regional lymph nodes at the time of surgery; residual cancer burden (RCB) scores, calculated according to the RCB index proposed by Symmans et al [16]; rate of breast-conserving surgery (BCS).

This study was approved by the local Ethical Review Board of IJB and it was conducted in compliance with Good Clinical Practices and the Helsinki Declaration. All patients provided written informed consent before entering the study.

### Treatment Planning and Response Assessments

EndoTAG-1 (22 mg/m^2^) and paclitaxel (70 mg/m^2^) were administered at weekly intravenous (i.v.) infusions for 12 week on the same day. After this, three cycles of FEC100 regimen (Fluorouracil 500 mg/m^2^, Epirubicin 100 mg/m^2^, and Cyclophosphamide 500 mg/m^2^) were administered every three weeks. MRI assessment was performed before any given therapy and no more than three weeks after the last taxane administration before the first FEC administration.

The volume calculation for the MRI assessment was performed using a computer-assisted, threshold-based segmentation algorithm, user-correctable (Telemis Inc., Louvain-la-Neuve, Belgium) on subtraction images from a 3D sequence with a 2 mm slice thickness and a 448x336 matrix size. The computer-aided, user-controlled segmentation algorithm reduces inter-observer variability. In addition, data were acquired on a 3D sequence which is nearly isometric and the segmentation algorithm is 3D, voxel-based, so no extrapolation was done using empirical formula. Segmentation on subtracted images allows avoiding difficult segmentation problems with fat or other high-signal tissue. The radiologist was not blinded to treatment.

### Statistical Analysis

We planned a sample size of 20 patients, based on a one-sided t-test for the average percentage decrease in MRI-estimated volume at the end of EndoTAG-1/Paclitaxel treatment from baseline. The null hypothesis was that the EndoTAG-1/Paclitaxel combination has no or a negligible effect on volume reduction, defined as a decrease ≤50% from baseline, whereas the alternative hypothesis was that this combination would yield at least an 80% average decrease. A standard deviation estimate of 59% was obtained from the study of Delille JP et al, a multiregimen neoadjuvant chemotherapy study that included 14 patients and for which the average percent decrease was equal to 60% [17]. For a one-sided significance level of 0.1 and a power of 82%, at least 20 patients were required. Due to skewness in the distribution of the variables MRI-estimated tumor volume, MRI-measured greatest diameter and MRI-estimated Ktrans tumor perfusion, we have applied the nonparametric sign test to compare the baseline with the end of treatment values.

## Role of the Funding Source

IJB was the sponsor of this study and Medigene AG provided the EndoTAG-1 and an educational grant.

## Results

### Patients

**Fig 1:**
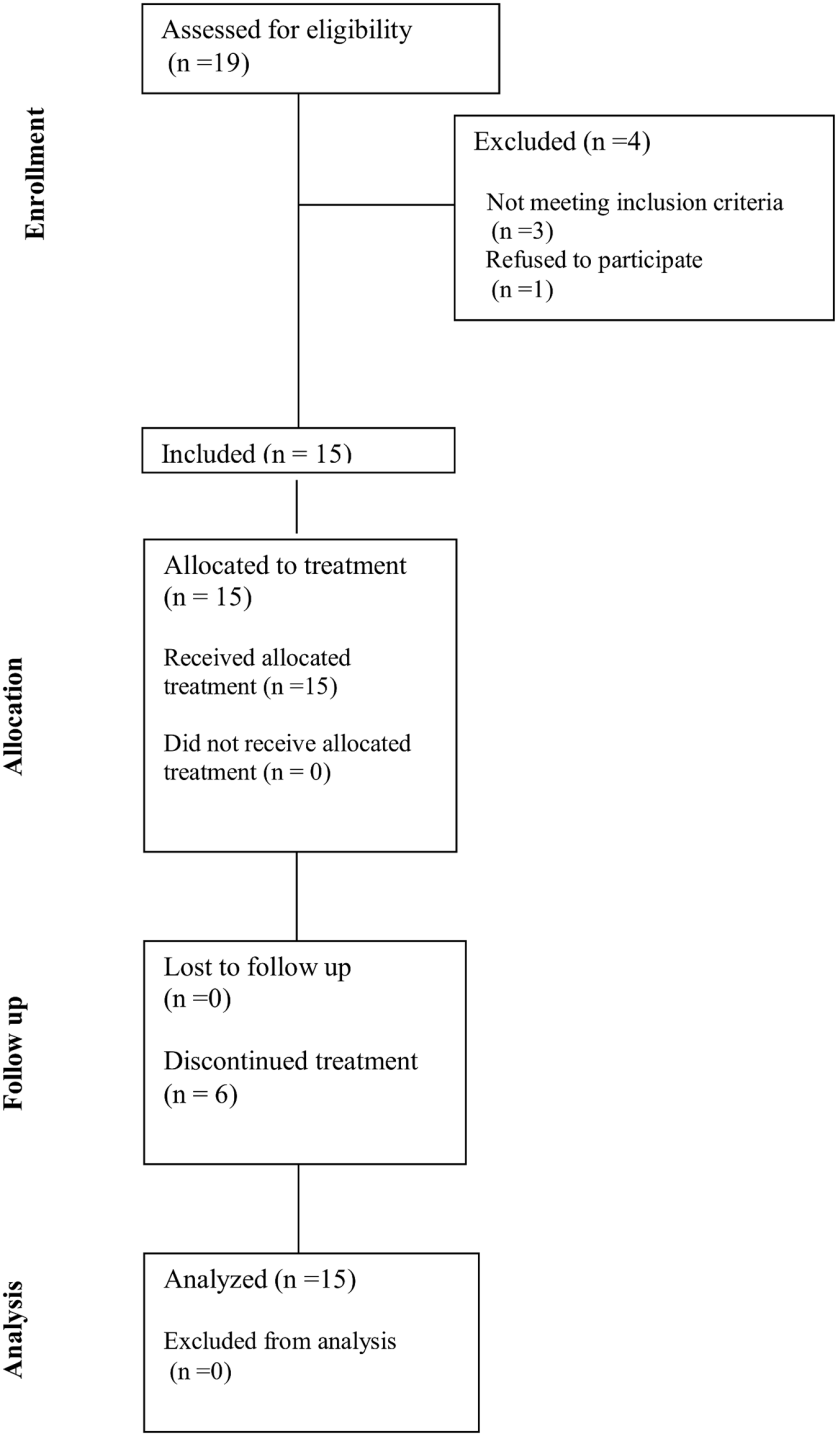
CONSORT Diagram.

A total of 15 patients were enrolled in this study, between December 2011 and May 2012 (Fig 1). The study did not reach the planned sample size because of safety concerns, as reported in the safety section. Six of these patients did not complete the initially planned number of treatments. Patient characteristics are summarized in Table 1. Median age was 47 years (range 29–63 years), 67% of the patients were premenopausal, 53% had a histological diagnosis of ductal carcinoma, 40% had tumors of histology Grade III and 80% of them had Ki67 ≥14%. Nine patients had ER-positive/HER2-negative BC and 6 had TNBC.

**Table 1:** Patient and disease characteristics at baseline.

Characteristic	No of Patients	%
**Age, years**		
Median	47	
Range	29–63	
**Menopausal Status**		
Premenopausal	10	67%
Postmenopausal	2	13%
Missing	3	20%
**Histology**		
Ductal	12	80%
Lobular	2	13.3%
Mixed (Ductal and Lobular)	1	6.7%
**ER/PR**		
ER+/PR+	8	53.3%
ER+/PR-	1	6.7%
ER-/PR+	0	0%
ER-/PR-	6	40%
**Ki67**		
<14	3	20%
≥14	12	80%
**Grade**		
I	1	7%
II	8	53%
III	6	40%
**cT**		
T1	3	20%
T2	8	53.3%
T3	1	7%
**cN**		
N0	8	53.3%
N1	7	46.7%
**Type of Treatment**		
**Radiotherapy**		
No	-	-
Yes	15	100%
**Adjuvant Hormonal Therapy**		
No	6	40%
Yes	9	60%
**Adjuvant Chemotherapy**		
No	15	100%
Yes	-	-

### Safety

AEs of grade 3 to 4 occurred in eight (53%) of the 15 patients, with six (40%) of them attributed to the EndoTAG-1/Paclitaxel chemotherapy (Table 2). Two patients experienced serious AEs: one grade 3 drug-hypersensitivity, related to the EndoTAG-1/Paclitaxel treatment and one grade 4 neutropenia, which was associated with the FEC treatment. Despite the premedication administered and the slow infusion rates, four patients experienced grade 3 hypersensitivity to EndoTAG-1 during the 1^st^, 2^nd^, 3^rd^ and 6^th^ weekly infusion respectively and required permanent discontinuation of the EndoTAG-1. The patients that have experienced grade 3 hypersensitivity to EndoTAG-1 during the 1^st^, 3^rd^ and 6^th^ weekly infusion continued with paclitaxel alone in order to complete 12 cycles of treatment and then received 3 cycles of FEC100. The patient that has experienced a grade 3 hypersensitivity reaction during the 2^nd^ infusion continued with 3 cycles of FEC100 every 3 weeks and 3 cycles of docetaxel 100mg/m^2^ every 3 weeks. These grade 3 hypersensitivity events led to premature closure of patients' enrolment, before reaching the initially planned sample size of 20 patients. No fatal AE has been reported.

**Table 2:** Adverse events of grade 3 or 4.

Patient ID	Adverse Event	Grade	Serious	Relation to Study Medication
2	Febrile Neutropenia	4	No	FEC
2	Febrile Neutropenia	3	No	FEC
2	Anemia	3	No	FEC
2	Febrile Neutropenia	4	No	FEC
2	Neutropenia	3	No	FEC
5	Drug Hypersensitivity	3	Yes	EndoTAG-1/Paclitaxel
6	Drug Hypersensitivity	3	No	EndoTAG-1/Paclitaxel
7	Hepatic Enzyme Elevation	3	No	EndoTAG-1/Paclitaxel
9	Drug Hypersensitivity	3	No	EndoTAG-1/Paclitaxel
9	Neutropenia	3	No	EndoTAG-1/Paclitaxel
9	Neutropenia	4	No	FEC
10	Hepatic Enzyme Elevation	3	No	EndoTAG-1/Paclitaxel
10	Mucosal Inflammation	3	No	FEC
11	Drug Hypersensitivity	3	No	EndoTAG-1/Paclitaxel
12	Neutropenia	4	Yes	FEC

**Abbreviations**: FEC: Fluorouracil, Epirubicin, Cyclophosphamide, ID: Identifier

### Efficacy

The efficacy data are shown collectively in Table 3. The MRI-estimated volume decreased from median 6.36 cm^3^ (min 1.56 to max 40.87) at baseline to median 0.36 cm^3^ (min 0 to max 20.26) at the end of treatment, which was a statistically significant decrease (P-value <0.001). The percent reduction in the MRI-estimated tumor volume was on average 81% (95% CI, 66% to 96%). A significant reduction in tumor size as defined by MRI-measured greatest diameter at the end of EndoTAG-1/Paclitaxel treatment was also noted (median 51%, p<0.001).

**Table 3:** Efficacy results.

Patient ID	Doses of EndoTAG-1/Paclitaxel	% Decrease in MRI-tumor volume	% Decrease in MRI-max diameter	% decrease in tumour perfusion (Ktrans)	pCR	RCB	
						Continuous	Class
1	12	-100.00	-100.00	-90.76	Yes	0	0
2	12	-95.28	-52.94	-9.03	No	1.641	2
3	12	-90.12	-66.18	NA	No	1.395	2
4	12	-83.85	-33.03	-43.92	No	Not assessed	Not assessed
5	1	-94.34	-45.92	-14.43	Yes	0	0
6	6	-87.34	-22.43	-42.42	Yes	0	0
7	6	-0.46	-0.29	-35.89	No	3.993	3
8	12	-99.38	-82.66	-11.53	Yes	0	0
9	2	-85.26	-35.53	-68.52	No	1.828	2
10	12	-68.62	-56.57	0.41	No	Not assessed	Not assessed
11	1	-50.43	-43.78	-30.97	No	3.718	3
12	12	-98.51	-51.09	-91.55	No	3.92	3
13	12	-100.00	-63.60	NA	Yes	0	0
14	12	-100.00	54.36	-86.21	No	1.687	2
15	9	-60.35	-23.20	-4.46	No	4.299	3

**Abbreviations**: ID: Identifier, MRI: Magnetic Resonance Imaging, NA: Not Assessed, pCR: pathologic Complete Response, RCB: Residual Cancer Burden

A significant percent reduction in MRI-estimated Ktrans tumor perfusion at the end of EndoTAG-1/Paclitaxel administration was also noted among 13 patients with available information about MRI-estimated Ktrans tumor perfusion (median change %, 36%, p<0.001). Eight (53%) patients underwent lumpectomy, and seven (47%) underwent mastectomy, with all 15 (100%) patients undergoing axillary node dissection. Five (33%) of the 15 patients enrolled in the study achieved pCR at the time of definite surgery, all of them having TNBC, thus translating into an 83% pCR rate among this BC phenotype. The patients achieving pCR received 1, 6, 12, 12, and 12 cycles of EndoTAG-1/Paclitaxel respectively as part of their NAC (see safety section). Of interest, per cent reduction of MRI-estimated tumor volume at the end of EndoTAG-1/Paclitaxel treatment was statistically associated with pCR (96% versus 73% for patient achieving and not achieving pCR respectively, p = 0.04). Lastly, a strong inverse correlation was noted between RCB seen as a continuous variable and the MRI-estimated change in tumour volume at the end of EndoTAG-1/Paclitaxel treatment (Pearson correlation r = -0.66, p = 0.01) among 13 evaluable patients.

## Discussion

In this phase II study, we evaluated the feasibility of EndoTAG-1/Paclitaxel NAC in HER2 negative BC patients, followed by three cycles of FEC100. This regimen showed promising antitumor activity: EndoTAG-1/Paclitaxel NAC resulted in significant percent reduction in MRI estimated tumor volume and significant reduction in tumor size as defined by MRI-measured greatest diameter at the end of EndoTAG-1/Paclitaxel treatment as compared to baseline (p<0.001 both). Additionally, among 13 evaluable patients a significant percent reduction in MRI-estimated Ktrans tumor perfusion parameter at the end of EndoTAG-1/Paclitaxel administration was also noted (p<0.001).

A pCR rate of 33% was achieved overall, with five out of six (83%) TNBC cases achieving pCR at definitive surgery. However, due to the small sample size of our study, results should be interpreted with particular caution. Based on results from three published meta-analyses, there is a strong association between pCR and clinical outcome for BC patients receiving NAC [10, 18, 19].

One interesting finding from this study is the significant association between percent MRI-assessed tumor volume reduction at the end of EndoTAG-1/Paclitaxel administration and pCR. Our study confirms results from previous studies demonstrating that assessment of treatment response using MRI during NAC is associated with pCR in BC patients. In the I-SPY trial, among 216 women receiving NAC, MRI assessment performed during treatment (after one cycle of anthracycline-based regimen and between the anthracycline-based and taxane regimen) was found to be a stronger predictor of pCR than clinical assessment [20]. MRI-assessed tumor volume proved to provide the most accurate prediction for pCR. Another study, assessing MRI response monitoring during NAC (4 different regimens used, with all HER2-positive cases treated with trastuzumab-based regimens), showed that this represents an accurate tool in TNBC or HER2-positive BC (n = 85), but not in ER-positive/HER2-negative subtype (n = 103) [13]. Indeed, the change in MRI-assessed change in largest tumor diameter between baseline and during NAC was a significant predictor of residual disease at surgery for these two BC subtypes. Evidence associating pCR with treatment response assessed through MRI at the end of NAC is also available. A multi-center, retrospective analysis of 746 BC patients receiving NAC assessed the ability of MRI at the end of treatment to predict pCR in the breast: an overall accuracy of 74% was found with variances among different BC subtypes [21]. In particular, higher accuracy was noted for patients with TNBC and HER2-positive BC (negative predictive value of 60% and 62% respectively) in comparison to the luminal cases, probably influenced by the different pCR rates seen among these different subtypes.

In our study, MRI assessment was performed after the completion of the EndoTAG-1/Paclitaxel part of NAC and before the completion of all NAC. In the phase III, randomized GeparTrio trial, early radiologic assessment of tumor response, was performed using ultrasound [22]. Patients not responding to two initial cycles of NAC were randomly assigned to either continuation of the same regimen (n = 321) or to an alternative treatment (n = 301), with no improvement in the pCR (pCR 5.3% versus 6.0% respectively). To our knowledge, there has been no randomized trial assessing MRI early on as a tool to guide treatment selection in the neoadjuvant setting. This might be further explored in appropriately designed clinical trials [23].

In terms of safety, it should be noted that 4 patients experienced grade 3 hypersensitivity reactions to EndoTAG-1 that led to permanent discontinuation of EndoTAG-1. Additionally, AEs that were observed during the subsequent chemotherapy following the EndoTAG-1 administration might have not occurred for some patients at least. Due to the drug hypersensitivity reactions, this trial had to be suspended. This study showed promising antitumor activity of EndoTAG-1/Paclitaxel followed by FEC chemotherapy in the neoadjuvant setting of HER2-negative BC. Significant reductions in MRI-assessed tumor volume and maximum diameter were seen, with high pCR rate within the subpopulation of TNBC patients. A significant association was found between percent reduction of MRI-assessed tumor volume after EndoTAG-1/Paclitaxel NAC with both pCR and RCB. If the issue with hypersensitivity reaction is resolved, further clinical development of this regimen should be pursued in TNBC patients.

## Supporting Information

S1 Fig TREND checklist for the ‘Feasibility study of EndoTAG-1, a tumor endothelial targeting agent, in combination with paclitaxel followed by FEC as induction therapy in HER2-negative breast cancer’ study. (PDF)

S1 Text Protocol for the ‘Feasibility study of EndoTAG-1, a tumor endothelial targeting agent, in combination with paclitaxel followed by FEC as induction therapy in HER2-negative breast cancer’ study. (PDF)
