# Supplementary material for: Feasibility Study of EndoTAG-1, a Tumor Endothelial Targeting Agent, in Combination with Paclitaxel followed by FEC as Induction Therapy in HER2-Negative Breast Cancer
Source: PLoS One. 2016 Jul 25;11(7):e0154009. doi: 10.1371/journal.pone.0154009 (PMC4959730; doi:10.1371/journal.pone.0154009)
Supplement: S1 Text — (PDF) [file pone.0154009.s002.pdf]

FINAL VERSION

AN OPEN-LABEL PHASE II TRIAL EVALUATING THE EFFICACY AND SAFETY OF NEOADJUVANT ENDOTAG-1 IN COMBINATION WITH PACLITAXEL IN PATIENTS WITH HER2-NEGATIVE BREAST CANCER

Jules Bordet Institute drafting committee:

Dr Ahmad Awada
Dr Michail Ignatiadis
Dr Tatiana Besse Hammer
Dr Marc Lemort
Dr Stefan Michiels

Principal Investigator

Michail Ignatiadis MD, PhD

Ahmad Awada MD, PhD

The present document contains confidential information belonging to the Jules Bordet Institute.

September 6, 2011

Synopsis

Title	An open-label phase II trial evaluating the efficacy and safety of neoadjuvant EndoTAGTM-1 in combination with paclitaxel in patients with Her2-negative high risk breast cancer
Study code	IJBNeoEndoTAG-1
EudaCT Number	2011-002985-19
Sponsor	Medical Oncology Clinic<br>Breast Cancer Translational Research Laboratory<br>Institut Jules Bordet<br>Héger Bordetstraat 1<br>1000 Brussels, Belgium
Principal Investigator	A. Awada, MD, PhD / Michail Ignatiadis MD, PhD<br>Institut Jules Bordet, Brussels, Belgium
Clinical phase	Phase II
Study rationale	Breast cancer (BC) is the most frequently diagnosed cancer and the leading cause of cancer death among females, accounting for 23% of the total cancer cases and 14% of the cancer deaths worldwide in 2008 (Jemal et al 2011). With 89.9%, incidence rates are highest in Western Europe (Globocan 2008).<br><br>Approximately 80% of breast cancers do not express the human epidermal growth factor receptor 2 (Her2), including hormone receptor-positive and triple-negative tumors. Her2-negative breast cancers are, or eventually become, resistant to existing cytotoxic treatments and hormones therapies and are therefore associated with poorer outcomes than Her2-positive breast cancer (Damasceno 2011).<br><br>Preoperative (neoadjuvant) chemotherapy is used to improve surgical options for BC. According to the tumor's receptor status (estrogen, progesterone and Her2 receptors) individual chemotherapy is selected. Response to neoadjuvant chemotherapy has been used to predict outcomes. Patients whose tumors show pathological complete response (pCR) at surgery have better clinical outcomes including longer relapse-free survival (RFS) (Kong et al 2011).<br><br>A meta-analysis including 6402 patients with early breast cancer receiving anthracycline-taxane +/- trastuzumab containing neoadjuvant

chemotherapy revealed that 11.3% of 3890 patients with hormone receptor (HR)-positive breast cancer experienced pCR compared to 34.6% of 1912 patients with estrogen receptor (ER)-negative disease (von Minckwitz G et al SABCS 2008) . In a meta-analysis of gene expression profiling data from 788 patients treated with anthracycline ± taxane-based neoadjuvant chemotherapy, pCR was reported in 112(30.1%) of 372 with ER-/HER2-, 41(36%) of 113 with ER-/HER2+, and 25(8.2%) of 303 patients with ER+/HER2- breast cancer (Ignatiadis et al unpublished data). After a median follow-up time of 3.47 years (95% CI 3.18-3.70) in the same study, patients with pCR had significantly longer RFS irrespective of BC subtypes (HR=0.20, 95% CI 0.08 to 0.50, p<0.001).

Despite these options for systemic adjuvant or neoadjuvant therapy, many patients with HER2-negative breast cancer relapse and die of the disease. There is a high medical need for novel (targeted) therapies that are appropriate to Her2-negative breast cancers and can improve clinical outcome.

EndoTAG^{TM}-1 is a cationic liposomal paclitaxel that has shown activity in triple-negative breast cancer (TNBC) when used in combination with standard paclitaxel. In a randomized controlled phase II study in TNBC, patients with 0-1 prior chemotherapy regimens for metastatic disease and a (neo)-adjuvant taxane free interval of > 6 months, the clinical benefit rate (complete or partial response at any time and stable disease ≥ 6 months) on combination of EndoTAGTM-1 and paclitaxel (n=50 patients) was 53% compared to 31 and 36% on EndoTAGTM-1 and paclitaxel monotherapy, respectively (Awada et al SABCS 2011). Safety analysis revealed the known toxicities for EndoTAGTM-1 and paclitaxel. EndoTAGTM-1 showed a similar safety profile to paclitaxel. On combination treatment, a slight increase in grade 3/4 adverse events was observed compared to either monotherapy, with neutropenia being the most predominant adverse event.

Several studies have used magnetic resonance imaging in order to evaluate tumor volume and tumor perfusion reduction in women with breast cancer receiving neoadjuvant chemotherapy (Delille et al Radiology 2003, Baar et al CCR 2009Ah-See et al CCR 2008)

The present study will be conducted to investigate the EndoTAG^{TM}-1/paclitaxel combination therapy in a regimen followed by administration of Fluorouracil, Epirubicin and Cyclophosphamide (FEC) in patients

	eligible for neoadjuvant chemotherapy in Her2-negative BC.
Study design	This is a prospective, single-center, open-label phase II clinical trial investigating the the activity of EndoTAGTM-1 + paclitaxel combination therapy in patients with Her2-negative BC candidate for neoadjuvant chemotherapy, as measured by the decrease in MRI-estimated tumour volume at the end of EndoTAGTM-1 + paclitaxel administration. Patients will be stratified by hormone receptor status.<br><br>A total of 20 female patients with non-metastatic Her2-negative breast cancer candidate for neoadjuvant chemotherapy and meeting all study eligibility criteria will be administered:<br><br>• 12 weekly infusions of EndoTAGTM-1 (22 mg/m2 liposomal paclitaxel) in combination with paclitaxel (70 mg/m2) followed by 3 cycles of FEC (Fluorouracil 500mg/m2, Epirubicin 100mg/m2, Cyclophosphamide 500mg/m2) every 3 weeks (experimental group)<br><br>The descriptive statistical analysis will be performed following the surgery of the last randomized patient.<br><br>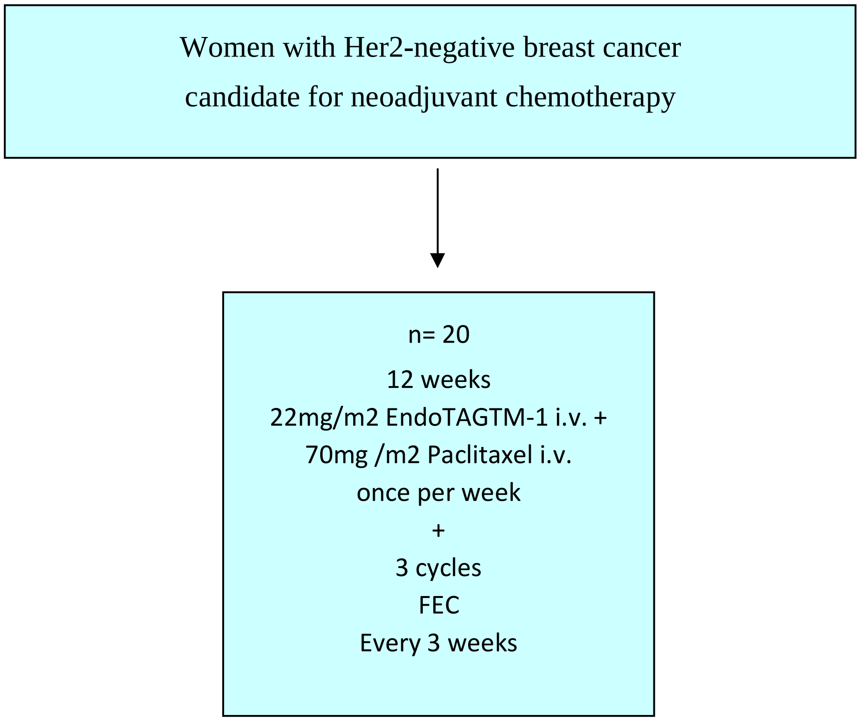

Objectives	**Primary objective:**<br><br>To investigate the activity of EndoTAGTM-1 + paclitaxel combination therapy in patients with Her2-negative BC candidate for neoadjuvant chemotherapy, as measured by the decrease in MRI-estimated tumour volume at the end of EndoTAGTM-1 + paclitaxel administration vs. baseline.<br><br>**Secondary objectives:**<br><br>To evaluate:<br>• Reduction in tumor size as defined by MRI-measured greatest diameter(s) following RECIST criteria at the end of EndoTAGTM-1 + paclitaxel administration<br>• Decrease in MRI-estimated tumor perfusion at the end of EndoTAGTM-1 + paclitaxel administration<br>• Pathological complete response (pCR)<br>• Rate of residual cancer burden at time of surgery<br>• Safety and tolerability of the neoadjuvant regimen<br>• Rate of breast-conserving surgery (BCS)<br>• Rate of clinical complete and partial responses (CR, PR)<br>• Rate of lymph node-negative disease after treatment (node-NEG)<br>To collect frozen tumor biopsies at baseline and surgery for future translational research on DNA and RNA
Endpoints	**<u>Primary efficacy endpoint:</u>**<br><br>**The percent reduction in MRI-estimated tumor volume at** the end of

	EndoTAGTM-1 + paclitaxel administration vs. baseline.<br><br>**Secondary efficacy endpoints:**<br><br>• **The percent reduction in linear tumour size as measured on MRI** by the greatest linear diameter(s) of the tumour following RECIST criteria at the end of EndoTAGTM-1 + paclitaxel administration<br>• **The percent reduction in** MRI-estimated tumour perfusion at the end of EndoTAGTM-1 + paclitaxel administration<br>• **Pathological complete response** (pCR) is defined as the absence of any residual invasive cancer in the breast and the absence of any metastatic cells in the regional lymph nodes at the time of surgery (University of Texas MD Anderson Cancer Center trial's pCR criteria)<br>• **Residual cancer burden** (RCB) scores are calculated according to the RCB index proposed by Symmans et al (2007)<br>• Rate of breast-conserving surgery (BCS)<br>• Rate of clinical complete and partial responses (CR, PR)<br>• Rate of lymph node-negative disease after treatment (node-NEG)<br>•<br><br>**Safety endpoints:**<br><br>• **Adverse Events (AEs)**: Incidence and percentage of patients with treatment emergent AEs<br>• **Laboratory Values:** Number of clinically significant abnormal laboratory values<br>• **Dose variations:** Percentage of patients having dose reductions, delays or discontinuation of study medication<br>•
Number of subjects	**20 women** with Her2-negative BC candidate for neoadjuvant chemotherapy<br><br>The required number of patients is based on the following assumptions: The sample size was based on a one-sided t-test for the average percentage decrease in MRI- estimated volume at surgery from baseline The null hypothesis is that combination has no or a negligible effect on

	volume reduction (defined as lower or equal to a 50% decrease) versus the alternative hypothesis that the new drug yields at least a 80% average decrease. A standard deviation estimate of 59% was obtained from Delille et al 2003, a multiregimen neoadjuvant chemotherapy study that included 14 patients and for which the average percent decrease was equal to 60%. For a one-sided significance level of 0.1 and a power of 82%, at least 20 evaluable patients are required.
Inclusion criteria	1. newly diagnosed histologically confirmed BC with breast infiltrating carcinoma of histological grade > 1 (either operable, or locally advanced or inflammatory) candidate for neoadjuvant chemotherapy<br>2. Her2-negative tumor, defined according to immunohistochemistry or using fluorescent in-situ hybridization (FISH)<br>3. ECOG performance status 0 or 1<br>4. Gender: female<br>5. Age ≥ 18 years old<br>6. Negative pregnancy test (females of childbearing potential)<br>7. Willingness to perform double-barrier-contraception during study and for 6 months post chemotherapy treatment (females of childbearing potential)<br>8. Signed informed consent
Exclusion criteria	1. Metastatic or relapsed disease<br>2. Major surgery < 3 weeks prior to enrollment<br>3. Severe pulmonary obstructive or restrictive disease<br>4. Uncontrolled inflammatory disease (autoimmune or infectious)<br>5. Clinically significant cardiac disease (NYHA stadium > 2)<br>6. Results of laboratory tests (hematology, chemistry) outside specified limits:<br>• WBC ≤ 3 x 10^9/L<br>• ANC < 1.5 x 10^9/L<br>• Platelets < 100 x 10^9/L<br>• Hb ≤ 9.0 g/dl (≤ 5.6 mmol/l)<br>• PTT/ INR > 1.5 x ULN

	• AST or ALT > 2.5 x ULN<br>• Alkaline Phosphatase > 2 x ULN<br>• Total Bilirubin > 1.5 x ULN<br>7. Pregnancy or nursing status<br>8. Known positive HIV testing<br>9. Known hypersensitivity to any component of the EndoTAG^{TM}-1, taxane or FEC formulations<br>10. History of malignancy other than breast cancer < 5 years prior to enrollment, except skin cancer (i.e. basal or squamous cell carcinoma) treated locally<br>11. History of active or significant neurological disorder or psychiatric disorder that would prohibit the understanding and giving of informed consent, or would interfere in the clinical and radiological evaluation of central nervous system during the trial<br>12. Concurrent treatment with other experimental products. Participation in another clinical trial with any investigational product within 30 days prior to study entry
Investigational medicinal product(s)	EndoTAG^{TM}-1: cationic liposomal membranes embedded with paclitaxel, will be provided by MediGene AG, Planegg / Martinsried, Germany.
Treatment	**Combination chemotherapy**<br><br>EndoTAG^{TM}-1 (<u>22 mg/m^2 liposomal paclitaxel)</u> + Paclitaxel (<u>70 mg/m^2</u>)<br>Weekly i.v. infusions of EndoTAG^{TM}-1 and paclitaxel for 12 weeks<br>• EndoTAG^{TM}-1 is given prior to paclitaxel on the same day<br>• EndoTAG^{TM}-1 infusion should be started slowly and increased to a maximum speed of 1.5 ml/min (15 min at 0.5 ml/min, 15 min at 1.0 ml/min and thereafter 1.5 ml/min)<br>• Paclitaxel infusion is given within 1h according to the manufacturer's instructions
Criteria for evaluation	MRI-estimated tumour volume and perfusion<br><u>Pathological complete response:</u><br>pCR will be evaluated at the time of surgery according to University of Texas MD Anderson Cancer Center trial's pCR criteria.

	- Residual cancer burden scores are calculated according to the RCB index proposed by Symmans et al (2007)<br><br><u>Safety evaluations:</u><br>- Adverse Events<br>- Safety laboratory variables (hematology, coagulation and clinical chemistry)<br>- Dose reductions, dose pausing and discontinuations of EndoTAG^{TM}-1 or paclitaxel<br>- Physical and neurological examination<br>- Vital signs
Statistical methods	The efficacy and safety analyses will be performed in the intent-to-treat (ITT) population. The ITT population consists of all randomized patients who had at least one administration of study medication.<br><br>The primary analysis will consist of a one-sided t-test at the 0.1 significance level.<br><br>The efficacy and safety analyses will be also performed in the per-protocol (PP) population. The PP population consists of all patients in the ITT population treated according to the procedures of the protocol without any protocol violations.<br><br>Continuous variables will be summarized using standard summary statistics as appropriate (n, mean, standard deviation, median, minimum, maximum, 25th and 75th percentiles). Summary statistics for categorical variables will include frequency counts and percentages, together with 90% confidence intervals.<br><br>Subgroup analyses may be included to evaluate efficacy and safety in patients according to hormone receptor status..
Number of sites	1
Time schedule	FPI (1st patient in): Sept 2011<br>Last possible treatment with EndoTAG-1 : 31.July 2012; therefore LPI (last patient in): 30. April 2012<br>LPO (last patient out): Nov 2012
End of Study	LPO (last patient out) = last patient enrolled having performed post-surgery visit for adjuvant therapy planning

INDEX

1.0 RATIONALE

2.0 STUDY DESIGN
- **2.1 OVERALL STUDY DESIGN AND PLAN**
- **2.2 TRIAL PERIODS**
- **2.3 STUDY OBJECTIVES**
- **2.4 STUDY ENDPOINTS**

3.0 STUDY HYPOTHESIS

4.0 PATIENT POPULATION / ELIGIBILITY CRITERIA
- **4.1 INCLUSION CRITERIA**
- **4.2 EXCLUSION CRITERIA**

5.0 EXAMINATIONS
- **5.1 PRE-TREATMENT EXAMINATION AND TUMOR STAGING**
- **5.2 EXAMINATIONS DURING NEOADJUVANT CHEMOTHERAPY**

6.0 TREATMENT OF PATIENTS
- **6.1 ENDOTAG™-1/PACLITAXEL REGIMEN**
- **6.2 FEC REGIMEN**

7.0 DOSE-MODIFICATION, DELAY, OR WITHDRAWAL
- **7.1 DOSE MODIFICATION**
- **7.2 DOSE DELAY**
- **7.3 TREATMENT WITHDRAWAL**
- **7.4 WITHDRAWAL FROM STUDY PARTICIPATION**

8.0 BREAST CANCER SURGERY AND POST-OPERATIVE TREATMENTS

9.0 EVALUATIONS AND PROCEDURES
- **9.1 ASSESSMENT OF EFFICACY**
- **9.2 ASSESSMENT OF SAFETY**
- **9.3 TRANSLATIONAL ASSESSMENTS**

10.0 FOLLOW-UP

11.0 STATISTICS

12.0 STUDY REGISTRATION AND LOGISTICS

13.0 ETHICS, AUTHORITIES AND INFORMED CONSENT

14.0 DOCUMENTATION
- **14.1 ADVERSE EVENT RECORDING AND REPORTING**
- **14.2 CASE REPORT FORMS**
- **14.3 DATA MANAGEMENT**
- **14.4 STATISTICAL ANALYSIS**

15.0 PUBLICATION POLICY

16.0 APPENDICES

1.0 RATIONALE

1) **Neoadjuvant chemotherapy for early breast cancer**

Breast cancer (BC) is the most frequently diagnosed cancer and the leading cause of cancer death among females, accounting for 23% of the total cancer cases and 14% of the cancer deaths worldwide in 2008 (Jemal et al 2011). With 89.9%, incidence rates are highest in Western Europe (Globocan 2008).

As a result of early detection through mammography and of improved treatment, BC death rates have been decreasing over the past 25 years (Jemal et al 2011). Due to screening programs, a growing number of tumors are detected in early, operable stages of the disease. Most patients have no or only few axillary lymph nodes involved and therefore a chance of being cured (Kantelhardt et al 2011). The administration of chemotherapy in addition to surgery has further increased disease-free and overall survival rates, with polychemotherapy being the preferred option of treatment. Adjuvant chemotherapy studies demonstrated improved disease-free and overall survival with anthracycline-based regimens such as FEC-100 (5FU, epirubicin and cyclophosphamide) (Bonneterre et al 2005). The combination of taxanes with anthracycline-based therapies has demonstrated added benefit (Bedard et al 2010).

Preoperative (neoadjuvant) chemotherapy is used to improve surgical options for BC. Recently, neoadjuvant therapy has become more commonly used in patients with larger tumors and/or lymph node involvement at diagnosis. Neoadjuvant chemotherapy reduces tumor burden, allowing the possibility of breast-conserving surgery, treats clinically undetectable micrometastases and allows for monitoring of tumor response to therapy (Hennessy et al 2006, Mieog et al 2007). Although no single chemotherapy regimen is specifically recommended, regimens tend to be anthracycline-based with the addition of taxanes, especially for high-risk, node-positive disease (Medioni et al 2011, Cuppone et al 2008).

In BC, subtypes can be defined according to the tumor's receptor status (estrogen, progesterone and Her2 receptors) using immunohistochemistry or cytogenetic techniques for classification. There is a clear association between BC subtype and patient outcomes. Response to neoadjuvant chemotherapy has been correlated with relapse and survival and might be used to predict outcomes. Patients whose tumors show pathological complete response (pCR) at surgery have better clinical outcomes including longer relapse-free survival (RFS) than patients with incomplete response (Kong et al 2011).

A meta-analysis (von Mickwitz et al SABCS 2008) including 6402 patients with early breast cancer receiving anthracycline-taxane +/- trastuzumab containing neoadjuvant chemotherapy revealed that 11.3% of 3890 patients with hormone receptor (HR)-positive breast cancer experienced pCR compared to 34.6% of 1912 patients with estrogen receptor (ER)-negative disease.

In a meta-analysis of gene expression profiling data from 788 patients treated with anthracycline ± taxane-based neoadjuvant chemotherapy, pCR was reported in 112(30.1%) of 372 with ER-/HER2-, 41(36%) of 113 with ER-/HER2+, and 25(8.2%) of 303 patients with ER+/HER2- breast cancer (Ignatiadis et al, unpublished data). After a median follow-up time of 3.47 years

(95% CI 3.18-3.70) in the same study, patients with pCR had significantly longer RFS irrespective of BC subtypes (HR=0.20, 95% CI 0.08 to 0.50, p<0.001).

Neoadjuvant anti-tumor activity of an alternating taxane- and anthracycline-based dose-dense regimen was investigated in patients with operable BC (Medioni et al 2011). The pCR rate was 22% overall, but was higher in triple receptor-negative BC (TNBC) than in patients without TNBC (40.9% versus 14.0%, respectively). Among the patients experiencing pCR, patients with TNBC had similar RFS and overall survival (OS) to patients without TNBC.

In a phase II trial, 8 cycles of docetaxel (75mg/m2, q3w) followed by FEC have been evaluated as neoadjuvant chemotherapy for patients with primary BC (Iwata et al 2011). The treatment regimen achieved high rates of pCR (23%) and an overall clinical response rate (ORR) of 79%.

Fisher et al (2011) reviewed data on patients with stage I-III TNBC in a retrospective analysis. Of 385 patients, 39% received neoadjuvant and 61% received adjuvant chemotherapy. 17% of the patients receiving neoadjuvant chemotherapy had pCR. An overall survival benefit was detected in patients with pCR following neoadjuvant chemotherapy compared to patients receiving adjuvant therapy.

Despite these options for systemic adjuvant or neoadjuvant therapy, many patients with HER2-negative breast cancer relapse and die of the disease. There is a high medical need for novel targeted therapies that are appropriate to Her2-negative breast cancers and can improve clinical outcome.

2) **EndoTAG^{TM}-1**

EndoTAG^{TM}-1 is an innovative vascular targeted therapy of cationic liposomal membranes embedded with paclitaxel. Cationic liposomes are known to bind and internalize at the negatively charged activated tumor-associated vascular endothelial cells after intravenous administration, forming the mechanistic basis for the new mode of action of EndoTAG-1. The cytostatic and cytotoxic activities of paclitaxel are thus delivered directly to tumor-associated activated endothelial cells, conferring, in addition to the known antitumour effects of paclitaxel, unique tumour neovasculature disrupting properties to EndoTAG-1. Additionally, since activated endothelium is genetically distinct and more stable than tumour tissue, EndoTAG-1 is expected to disrupt neovasculature also in taxane-resistant tumours. EndoTAG- 1 is intended for the treatment of solid tumors.

The growth of tumors beyond a size of 1-2 mm requires the formation of new blood vessels, ensuring the flow of oxygen, growth factors, nutrients and other substances to the tumor tissue. Capillaries sprout, grow toward and infiltrate the tumor mass. The involved proliferating endothelial cells display anionic molecules at their surface, such as glycoproteins, anionic phospholipids and proteoglycans. Cationic liposomes have selective affinity for these anionic molecules, therefore EndoTAG^{TM}-1 is expected to bind to and accumulate in the tumor microvessels, where encapsulated paclitaxel exerts its cytotoxic effects on tumor endothelial cells (vascular targeting) (Abu Lila et al 2010, Eichhorn et al 2010, Campbell et al 2002).

Accordingly, cationic liposomes are able to extravasate into the tumor interstitium via the leaky tumor vasculature. The cytotoxic agent paclitaxel, encapsulated in these liposomes will be allowed to act on tumor cells as well (tumoral targeting) (Abu Lila et al 2010). In addition,

chemotherapeutic agents given in combination with EndoTAG^{TM}-1 might have easier access to tumor cells because of the leaky tumor vasculature. Finally, spreading of tumor cells through the bloodstream to distant sites will be abolished by destruction of tumor blood vessels.

Proliferating endothelial cells in solid tumors share similar phenotypes independent of the type of tumor or tumor location, therefore targeting of EndoTAG^{TM}-1 to tumor endothelium is possible in a wide variety of solid tumors. Since the route of application of EndoTAG^{TM}-1 is intravenous infusion, metastases of a primary tumor will automatically be treated in parallel, if formation of new blood vessels has been accomplished, irrespective whether metastases have been detected or not.

3) **Non-clinical studies using EndoTAG^{TM}-1**

EndoTAG^{TM}-1, when given intravenously in a variety of syngeneic and xenograft tumor models in mice, hamster and rats, showed a pronounced higher uptake into tumor tissue compared to normal tissue. In all models tested, EndoTAG^{TM}-1 led to a significantly reduced tumor growth activity compared to treatment with standard paclitaxel, independent of tumor type, animal species and immune status. In addition, the rate of metastasis was significantly decreased by EndoTAG^{TM}-1 compared to standard paclitaxel. Furthermore, EndoTAG^{TM}-1 was shown to be effective in paclitaxel-resistant tumors in animals.

An increased anti-tumor activity in animals was observed when EndoTAG^{TM}-1 was combined with other cytostatic or cytotoxic agents. Combination therapy (e.g. EndoTAG^{TM}-1 + gemcitabine) showed enhanced tumor regression, reduction of lymph node metastases and absence of liver metastases compared to either monotherapy. In vitro, combination therapies of EndoTAG^{TM}-1 with chemotherapeutics showed complete or concentration-dependent additivity, thus supporting the in vivo data.

Mode-of-action studies in rodents demonstrated the neovascular targeting properties of EndoTAG^{TM}-1. A strong decrease of the endothelial proliferation index in the tumor periphery as well as a reduction of the tumor perfusion index and an increase of endothelial cell apoptosis was observed. These results indicate a potential dual mode-of-action of EndoTAG^{TM}-1 by combining anti-neovascular and anti-tumor effects (Eichhorn et al 2010, Strieth et al 2008, Strieth et al 2004, Schmitt-Sody et al 2003).

Toxicology

The toxicological profile of EndoTAG^{TM}-1 has been evaluated in single- and repeated-dose toxicity studies in mice, rats and dogs covering treatment periods of up to 26 weeks.

In an acute toxicity study in mice mortality was observed at 5.4 mg/kg BW liposomal paclitaxel. The same mortality was reported in the control group using empty liposomes and was most likely due to the large volumes of injected suspended lipid complexes. No other findings were reported in the acute toxicity studies.

In subchronic toxicity studies in rats the high dose EndoTAG^{TM}-1 group (1.5 mg/kg BW liposomal paclitaxel) showed slight weight loss and reduction of white blood cells. The main

histopathological finding was hypocellularity of the hematopoetic bone marrow in about one third of the rats in this dose group. The effects were reversible and resolved during recovery.

Acute, subchronic and chronic repeated dose toxicity studies have been performed in dogs. In the 14-day and 28-day studies liposomal paclitaxel doses up to 1.7 mg/kg BW per application were given daily or every other day. At higher doses reduced food uptake accompanied by a moderate but continuous reduction in body weight was observed and lesions were found in the gastro-intestinal tract – findings which are attributed to the action of paclitaxel. With daily treatment, lethal toxicity was observed in all dose groups. When dosed at 2-day intervals, treatment was tolerated better and only some animals of the higher dose groups died. In the 90-day and 180-day studies, doses up to 1.2 mg/kg BW were applied twice a week, resulting in a total of 26 and 53 applications, respectively. Due to severe toxicities including mortality observed in the high dose group, treatment was interrupted and, in part, reduced to one application per week.

A "no observed adverse effect level" (NOAEL) could not be established in studies covering a treatment period of 3 or 6 months, which is common for cytostatic and cytotoxic drugs with low therapeutic index. The non-clinical toxicity studies indicate that the haematopoietic system, the gastrointestinal tract including the liver and the gall bladder and the male gonads are the potential target organs in humans. Microscopic lesions were seen mainly in areas of high tissue turn-over such as the gastrointestinal tract and the testes. Based on acute findings (hemorrhagic pulmonary edema) observed in a number of animals that died prematurely in the subchronic and chronic toxicity studies in dogs, the lung represents an additional target organ. During clinical application of EndoTAG^{TM}-1 the function of these organ systems should be closely monitored. Other known side effects of standard paclitaxel were not observed during administration of EndoTAG^{TM}-1.

Pharmacokinetics

The pharmacokinetic profile of EndoTAG^{TM}-1 has been established in single dose studies in mice and in repeated dose toxicokinetic studies in rats and dogs. The highest systemic exposure of paclitaxel from EndoTAG^{TM}-1 was observed in humans and dogs, and the lowest in rats and mice. These findings were in line with the clearance rates observed, which were low for non-rodents (humans/ dogs) and high for rodents (rats/ mice).

A linear pharmacokinetic relationship was determined for the administered EndoTAG^{TM}-1 dose and the C_{max} and AUC of paclitaxel in rats and dogs. The pharmacokinetic profiles of paclitaxel from EndoTAG^{TM}-1 compared to standard paclitaxel in rodents seemed to be almost identical at lower doses (1.1 mg/ kg BW liposomal paclitaxel), whereas at higher doses (5 mg/ kg BW liposomal paclitaxel) C_{max} and AUC values of paclitaxel from EndoTAG^{TM}-1 were markedly lower. No comparative studies have been performed in non-rodents.

In vitro and in vivo studies demonstrated comparable metabolism of paclitaxel for EndoTAG^{TM}-1 and standard paclitaxel. Both agents resulted in similar biodistribution patterns and kinetics in female rats. The highest concentration of paclitaxel was found in the liver, but marked concentrations were also observed in kidney, lung, heart, spleen and adrenal gland. Paclitaxel was mainly excreted in bile (~88% of dose) and excretion was almost completed after 72 hours for both EndoTAG^{TM}-1 and standard paclitaxel.

The pharmacokinetic profile of the cationic liposomal constituent DOTAP is characterized by a linear relationship between the administered EndoTAG™-1 dose and C_{max} and AUC. In contrast to paclitaxel a later C_{max} was observed for DOTAP. It appears to be slowly distributed and is incompletely cleared from systemic blood circulation. The results of the biodistribution study in female rats showed that a large amount of DOTAP is transiently distributed to the lungs and relatively high levels are maintained until a marked decrease of DOTAP occurred between 6-24 hours. In contrast, liver, kidneys and spleen showed a gradual increase of DOTAP levels up to 6-24 hours. DOTAP was mainly excreted in the urine (78 % of dose) and excretion occurred rather slowly. After 5 days, 9.2% of the dose was still present in the body. Consequently, accumulation of DOTAP was observed in the plasma of rats and dogs when EndoTAG™-1 was administered twice per week for 26 weeks, but was not associated with a notable increase in toxicities.

In vitro studies with human liver microsomes suggested either the absence of or only a weak Cytochrome P450 inhibition potential for DOTAP, whereas Cremophor EL, the excipient of standard paclitaxel, turned out to be a very potent Cytochrome P450 inhibitor.

4) Clinical studies using EndoTAG™-1

A total of 445 patients with a variety of solid tumors have been treated with EndoTAG™-1 during participation in clinical studies.

Nine clinical studies (phase I/II) were initiated by the company Munich Biotech AG (MBT), which originally started the development of EndoTAG™-1. Various doses (2.63 - 66 mg/m² liposomal paclitaxel), infusion speeds (up to 2.5 ml/min) and schedules (daily to weekly, for up to nine weeks) were examined in these studies. In these studies, a total of 163 patients were treated with EndoTAG™-1. EndoTAG™-1 was usually given as a monotherapy except for one study in NSCLC (combination with carboplatin) and one study in gastrointestinal cancer (combination with 5-FU).

At MediGene AG, three phase II trials have been conducted:

- CT 4001 was a controlled, randomized, open label, multicenter trial in 200 patients with locally advanced or metastatic pancreatic cancer. The objective of this study was to evaluate safety and efficacy of a combination treatment with twice weekly EndoTAG™-1 at three dose levels (11, 22 and 44 mg/m²) and weekly gemcitabine (1000 mg/m²) compared to weekly gemcitabine alone (1000 mg/m²). 50 patients were randomized and treated in each study arm.
- CT 4002 was a controlled, randomized, open label, multicenter trial in 135 patients with relapsed and/or metastatic triple receptor-negative breast cancer. The objective of this study was to assess the safety and efficacy of a combination treatment with weekly EndoTAG™-1 (22 mg/m²) and standard paclitaxel (70 mg/m²) compared to either EndoTAG™-1 44 mg/m²) twice per week or standard paclitaxel alone (90 mg/m² weekly). Patients were randomized in a 2:2:1 ratio, respectively.
- CT 4003 was an uncontrolled, open-label, single site trial in 20 patients with hepatic metastases from a carcinomatous primary tumor other than hepatocellular (HCC), biliary or bile duct carcinoma. The objective of this study was to evaluate the single-dose and

steady-state pharmacokinetics of twice weekly EndoTAG™-1 (22 mg/m^2) and its effects on blood supply and angiogenesis of hepatic metastases.

Safety

Safety data of 163 patients suffering from various solid tumors are available from phase I/II studies. 138 (84.6 %) patients experienced one or more adverse events (AEs), 902 AEs were reported in total. Most of these events were assessed as being causally related to study medication by the investigator (668 of 902, or 74%) and were classified as non-serious (830 of 902, or 92%). The majority of these non-serious adverse drug reactions (ADR's) were of transient nature. Only 20 (2.2 %) AEs were assessed as serious and drug-related by the investigator.

In study CT 4001, AEs were reported in 92% to 100% of patients in each treatment arm. No unexpected toxicities have been observed. During the first treatment cycle, severe hematological toxicities (grade 3/4 NCI-CTC) related to any study medication occurred in 22, 32 and 40% of patients in the different GEM+Endo groups (11, 22 and 44 mg/m^2, respectively) compared to 24% on Gem monotherapy. Combination of EndoTAG™-1 and Gem resulted in a dose-dependent increase of grade 3/4 thrombocytopenia reaching up to 16% and 14% in the two higher dose levels, albeit without clinical symptoms or bleeding complications. At the highest EndoTAG™-1 dose level (44mg/m^2 twice weekly), increased rates of grade 3/4 neutropenia (22%) and anemia (12%) were observed.
During the first cycle, a total of 7 cases of febrile neutropenia were reported in the two higher GEM+Endo dose levels, including 4 cases of grade 3/4. During additional cycles with GEM+Endo therapy, none was observed.
Infusion-related reactions, predominantly pyrexia and chills, were found to a higher extent in GEM+Endo groups, whereas the addition of EndoTAG™-1 to Gem did not increase the known liver toxicity of Gem. In the GEM+Endo44 arm, one case of neuropathy was reported in a patient with diabetes, but was considered unrelated to study medication.
AEs resulting in discontinuation of study medication were reported in 4 patients (8%) in the GEM+Endo11 and 7 patients (14%) in each of the GEM+Endo22 and GEM+Endo44 group. During the first cycle, 2 patients (4%) in each of the GEM+Endo11 and GEM+Endo22 group, and 1 patient (2%) in the GEM+Endo44 group died, but deaths were considered not related to study medication. During additional cycles, another 2 patients of the GEM+Endo11 group had SAEs with fatal outcome. Both events, Staphylococcal sepsis and death from unknown cause, were considered unlikely to be related to study medication.

In study CT4002 safety analysis revealed qualitatively and quantitatively the known toxicities of EndoTAG™-1 and standard paclitaxel. AEs were reported in 91% to 100% of patients in each treatment arm. No unexpected toxicities have been observed. EndoTAG™-1 showed a similar safety profile to paclitaxel. On combination treatment, a slight increase in grade 3/4 adverse events was observed compared to either monotherapy, with uncomplicated neutropenia being the most predominant adverse event. Neutropenia was highest among patients during combination therapy (40%), whereas anemia was predominantly observed in the standard paclitaxel study arm (36%). Grade 3/4 hematological toxicities, especially neutropenia and leucopenia, occurred more often in the combination arm (20% and 7%) compared to either agent alone (4% and 2% for EndoTAG™-1 monotherapy, 7% and 0% for standard paclitaxel, respectively) (Awada et al, publication in preparation).

As was observed in CT4001, infusion-related reactions, predominantly pyrexia and chills were found to a higher extent in EndoTAG^{TM}-1 treatment arms and increased at higher doses. Peripheral sensory neuropathy was observed in 13% of the combination treatment arm compared to 9% and 14% of patients on EndoTAG^{TM}-1 monotherapy and standard paclitaxel, respectively. Severe neurotoxicity was reported for 1 patient (4%) of the standard paclitaxel treatment arm.

In study CT4003 all 20 patients experienced at least one AE, with a total of 330 AEs reported. 33 AEs of grade 3/4 and 21 SAEs were observed and reported from 11 (55%) patients. The most frequent AEs were abdominal pain, nausea, vomiting and fatigue in 10 to 14 patients (50-70%). Hypersensitivity and hyperhidrosis occurred in 8 patients each (40%) and were mostly of mild to moderate severity. Hematological toxicities were observed in 4 patients (20%), including 3 events of anemia and 1 event each of neutropenia and leucopenia (both of grade 3 severity). Peripheral sensory neuropathy was reported for 3 patients (15%). No AE with a severity of grade 4 was reported.

Pharmacokinetics

The pharmacokinetic properties of EndoTAG^{TM}-1 were determined in three phase I/II dose-escalation studies (CTLP01, CTLP05 and CTLP06) exploring several dose levels (2.63-66 mg/m² paclitaxel) and application regimen (2-5 times per week) and in study CT4003.

Results of studies CTLP01, CTLP05 and CTLP06 revealed that the maximum plasma concentrations and extents of exposure of paclitaxel and DOTAP, administered as EndoTAG^{TM}-1, increased with the dose. Data were too limited to assess whether the increase is proportional. The clearance of paclitaxel and DOTAP remained constant with increasing doses, with higher clearance levels observed for paclitaxel compared to DOTAP. There was no indication for paclitaxel accumulation in plasma at any dose level or application regimen studied. Although DOTAP is cleared from the plasma slowly, there was also no evidence for accumulation of DOTAP.

In study CT4003 reliable PK-profiles were obtained for paclitaxel and DOTAP after single dose and in steady state following intravenous infusion of EndoTAG^{TM}-1 (22 mg/m^2 twice weekly for 25 days). The pharmacokinetic parameters observed for paclitaxel and DOTAP confirmed previous results with 22 mg/m^2 EndoTAG^{TM}-1 from early clinical trials (CTLP05, CTLP06). The mean maximum plasma concentration of paclitaxel was comparable after single dose and in steady state. The extent of exposure showed a small increase from the first to the last dose. The mean maximum plasma concentration of DOTAP increased from the first to the last dose and the extent of exposure increased more pronounced compared to paclitaxel. Distribution and elimination kinetics of EndoTAG^{TM}-1 appear to be largely comparably to standard paclitaxel at lower dose levels. Only minor accumulation has been observed for paclitaxel and DOTAP, suggesting that no considerable saturation of distribution or elimination systems occurred. Finally, consistent gender-specific differences have not been observed with respect to the pharmacokinetics of paclitaxel and DOTAP (Fasol et al 2011).

Efficacy

In their phase I/II studies, Munich Biotech AG enclosed patients with hormone-refractory prostate cancer (CTLP01), unresectable locally advanced or metastatic gastrointestinal

(CTLP05) or colorectal cancer (CTLP10) and metastatic breast cancer (CTLP09) applying doses between 2.63 and 66 mg/m² liposomal paclitaxel in various regimen of up to 9 weeks treatment. Patients were evaluated for overall tumor response only, survival data were not collected. Complete responses were not reported. Partial responses were observed in 3 (8%, CTLP09) and 1 (3%, CTLP010) patients, stable disease was seen in 13 (36%, CTLP09), 3 (8%, CTLP010) and 4 (14%, CTLP05) of the patients. Accordingly the clinical benefit rate was 44% and 11% in CTLP09 and CTLP10, respectively. The effect on tumor stabilization was most distinct in patients with metastatic breast cancer (CTLP09) with a dose-dependent effect on the rate of patients with non-progressive disease (33% at 22 mg/m^2, 56% at 44 mg/m^2). In study CTLP01, PSA levels remained stable in the majority of patients during treatment, but increased during the follow-up period. 1/12 (8.33%) of patients showed stable disease (reduction of PSA levels > 50%) after 43 days.
In summary, disease stabilization and partial response has been observed in some of the patients. However, patient numbers were small and studies were conducted without comparator(s).

Phase II studies of MediGene AG were conducted in patients with advanced pancreatic cancer (CT4001), triple receptor-negative breast cancer (CT4002) and hepatic metastases from a carcinomatous primary tumor (CT4003).

In study CT4001, 3 different dose levels of EndoTAG^{TM}-1 (11, 22 and 44 mg/m^2 liposomal paclitaxel) in combination with gemcitabine (GEM+Endo) were compared to gemcitabine monotherapy (GEM) in 200 patients (50 patients in each study arm) with unresectable locally advanced or metastatic pancreatic adenocarcinoma. At the time of analysis 161 patients have died. Median overall survival was 7.2 months on GEM monotherapy compared to 8.4, 8.7 and 9.4 months in the GEM+Endo cohorts (11, 22 and 44 mg/m^2, respectively). Median PFS reached 2.7 months compared to 4.1, 4.6 and 4.4 months and the disease control rate after the first treatment cycle was 43% compared to 60, 65 and 52%, respectively. Accordingly, both the 6- and 12-month survival rates were higher in all GEM+Endo groups than in the GEM group (Löhr et al 2011, publication in press).

In study CT4002, 140 women with triple receptor negative metastatic or relapsed breast cancer have been randomized to 3 different study arms: EndoTAG^{TM}-1 (22 mg/m^2 liposomal paclitaxel) in combination with standard paclitaxel (70 mg/m^2), EndoTAG^{TM}-1 monotherapy (44 mg/m^2 liposomal paclitaxel, twice per week), and standard paclitaxel monotherapy (90 mg/m^2). Each treatment was given for 3 weeks, followed by 1 week of rest.
The disease control rate at week 16, after 4 cycles of therapy, was 59% on combination therapy compared to 34% in the EndoTAG^{TM}-1 and 48% in the standard paclitaxel cohort (Awada et al ESMO 2010). The clinical benefit rate (complete or partial response at any time and stable disease ≥ 6 months) on combination of EndoTAG^{TM}-1 and paclitaxel (n=50 patients) was 53% compared to 31 and 36% on EndoTAG^{TM}-1 and paclitaxel monotherapies, respectively (Awada et al SABCS 2011, submitted). PFS was 4.2 compared to 3.4 and 3.7 months, respectively. In the target population of the study, i.e. patients with TNBC, the overall survival was 13.0 months on combination of EndoTAG^{TM}-1 and paclitaxel versus 11.9 and 10.1 months on EndoTAG^{TM}-1 and paclitaxel monotherapies, respectively. Subgroup analysis in patients with advanced TNBC, ECOG performance status 0 or 1 and first line therapy revealed an overall survival of 17.8 months on combination of EndoTAG^{TM}-1 and paclitaxel versus 12.5 and 10.1 months on EndoTAG^{TM}-1 and paclitaxel monotherapies, respectively. However, the study was not powered for intergroup comparisons (Awada et al, publication in preparation).

Study CT4003 was conducted to evaluate pharmacokinetics of EndoTAG™-1 (22 mg/m^2 liposomal paclitaxel, twice a week) and its effect on blood supply and angiogenesis of hepatic metastases in 20 patients with a carcinomatous primary tumor other than hepatocellular, biliary or bile duct carcinoma. At the beginning of therapy all patients had stable disease according to modified RECIST 1.1 criteria. The median PFS was 29 days. The effect of EndoTAG™-1 on target liver metastases perfusion assessed by CEUS was inhomogeneous, inflow coefficients showed a great intra-individual variability and a trend could not be elaborated. However, changes in perfusion parameters assessed by DCE-MRI were observed. By Day 29, 65% of patients had decreases in ktrans, iAUC60, iAUC90 and iAUC120, supporting the hypothesis of a vascular targeting mode of action of EndoTAG™-1 (Fasol et al 2011).

In conclusion, EndoTAG™-1 has shown vascular targeting, vascular disrupting and anti-tumor activity in different tumors in several nonclinical and clinical studies. Therapeutic efficacy was highest when EndoTAG™-1 was administered in combination with other chemotherapeutics. In advanced TNBC, EndoTAG™-1 has shown benefit for the patients when used in combination with standard paclitaxel. The combination of EndoTAG™-1 and standard paclitaxel was well tolerated with uncomplicated neutropenia being the most predominant adverse event.

5) **Selection of patient population**

Approximately 80% of breast cancers do not express the human epidermal growth factor receptor 2 (Her2), including hormone receptor-positive and triple-negative tumors. Her2-negative breast cancers are, or eventually become, resistant to existing targeted treatments and hormones therapies and are therefore associated with poorer outcomes than Her2-positive breast cancer (Damasceno 2011). However, it has been shown that neoadjuvant chemotherapy improves survival in patients with Her2-negative tumors, if pCR was achieved at the time of surgery (Kong et al 2011, Medioni et al 2011, Iwata et al 2011, Fisher et al 2011, Ignatiadis et al, unpublished data). EndoTAG™-1 has shown anti-tumor activity in TNBC, when given in combination with standard paclitaxel. Treatment with this combination revealed the best results in patients receiving first-line therapy and having an ECOG performance status of 0 or 1.

For the present study, we therefore will select patients with chemotherapy-naïve, operable BC being available for neoadjuvant therapy and an ECOG performance status of 0 or 1. Patients should not have received previous treatment for BC and their tumors should have Her2-negative tumor status.

6) **Objectives and Purpose of the study**

The goal of neoadjuvant chemotherapy is to achieve pathological complete response (pCR) at time of surgery to increase chances for long survival or even cure of BC patients. Addition of taxanes to the FEC regimen has resulted in higher rates of pCR in patients with early breast cancer (Medioni et al 2011, Cuppone et al 2008). EndoTAG™-1 has improved the anti-tumor activity of standard paclitaxel in TNBC (Awada et al SABCS 2011 submitted, Awada et al ESMO 2010).

The present study will be conducted to investigate the EndoTAG^{TM}-1/paclitaxel combination therapy in a regimen followed by administration of Fluorouracil, Epirubicin and Cyclophosphamide (FEC) in patients eligible for neoadjuvant chemotherapy in chemotherapy-naïve, Her2-negative BC. Primary objective is the rate of patients with pCR at surgery. This study will investigate the activity of EndoTAGTM-1 + paclitaxel combination therapy in patients with Her2-negative BC candidate for neoadjuvant chemotherapy, as measured by the decrease in MRI-estimated tumour volume at the end of EndoTAGTM-1 + paclitaxel administration vs. baseline.

7) **References**

Abu Lila et al : Targeting anticancer drugs to tumor vasculature using cationic liposomes. Pharm Res 27: 1171-83, 2010.

Ah-See et al : Early changes in functional dynamic magnetic resonance imaging predict for pathologic response to neoadjuvant chemotherapy in primary breast cancer. Clin Cancer Research 14: 6580-6589 2008

Awada et al : Results of the first randomized phase II study of cationic liposomal paclitaxel (EndoTAG^{TM}-1) targeting tumor endothelial cells in advanced triple-negative breast cancer (TNBC). ESMO late-breaking abstract LBA12. Ann Oncol 21(suppl. 8): viii1-viii12, 2010.

Awada et al : Final results of a controlled, randomized 3-arm phase II trial of EndoTAG^{TM}-1, a cationic liposomal formulation of paclitaxel targeting tumor endothelial cells, in advanced triple-negative breast cancer (TNBC). SABCS 2011, abstract submitted.

Awada et al : CT4002, an open-label, randomized, controlled phase II trial evaluating the efficacy and safety of EndoTAG^{TM}-1 in triple receptor-negative breast cancer patients, publication in preparation.

Baar J et al : A vasculature-targeting regimen of preoperative docetaxel with or without bevacizumab for locally advanced breast cancer: impact on angiogenic biomarkers. Clin Cancer Res 15 3583-3590 2009

Bedard et al : Taxanes: optimizing adjuvant chemotherapy for early-stage breast cancer. Nat Rev Clin Oncol 1: 22-36, 2010.

Bonneterre et al : Epirubicin increases long-term survival in adjuvant chemotherapy of patients with poor prognosis, node-positive, early breast cancer: 10-year follow-up results of the French Adjuvant Study Group 05 randomized trial. J Clin Oncol 23: 2686-93, 2005.

Campbell et al : Cationic charge determines the distribution of liposomes between the vascular and extravascular compartments of tumors. Cancer Res 62: 6831-6, 2002.

Cuppone et al : Taxanes as primary chemotherapy for early breast cancer. Meta-analysis of randomized trials. Cancer 113: 238-46, 2008.

Damasceno M : Bevacizumab for the first-line treatment of human epidermal growth factor receptor 2-negative advanced breast cancer. Curr Opin Oncol 23 Suppl: S3-9, 2011.

Delille et al : Invasive ductal breast carcinoma response to neoadjuvant chemotherapy: noninvasive monitoring with functional MR imaging pilot study. Radiology 228: 63-69 2003

Eichhorn et al : Vascular targeting by EndoTAG-1 enhances therapeutic efficacy of conventional chemotherapy in lung and pancreatic cancer. Int J Cancer 126: 1235-45, 2010.

Fasol et al : Vascular and pharmacokinetic effects of EndoTAG-1 in patients with advanced cancer and liver metastasis. Ann Oncol Jun 21, 2011.

Ferlay et al : GLOBOCAN 2008 v1.2, cancer incidence and mortality worldwide: IARC CancerBase No. 10 (Internet). Lyon, France: International Agency for Research on Cancer; 2010.

Fisher CS et al : Neoadjuvant chemotherapy is associated with improved survival compared with adjuvant chemotherapy in patients with TNBC only after complete phathologic response. Ann Surg Oncol Jul2, 2011

Hennessy et al : Neoadjuvant therapy of breast cancer. Am J Cancer 5: 411-25, 2006.

Iwata H et al : Docetaxel followed by fluorouracil/epirubicin/cyclophosphamide as neoadjuvant chemotherapy for patients with primary breast cancer. Jpn J Clin Oncol 41(7): 867-75, 2011

Jemal A et al : Global Cancer Statistics. CA Cancer J Clin 61: 69-90, 2011

Kantelhardt et al : Prospective evaluation of prognostic factors uPA/PAI-1 in node-negative breast cancer: Phase III NNBC3-Europe trial (AGO, GBG, EORTC-PBG) comparing 6xFEC versus 3xFEC/3xdocetaxel. BMC Cancer 11: 140-9, 2011.

Kong X et al : Meta-analysis confirms achieving pathological complete response after neoadjuvant chemotherapy predicts favourable prognosis for breast cancer patients. Eur J Cancer Jul5, 2011

Löhr et al : Cationic liposomal paclitaxel plus gemcitabine or gemcitabine alone in patients with advanced pancreatic cancer: a randomized controlled phase II trial. Ann Oncol publication in press, 2011.

Mieog et al : Preoperative chemotherapy for women with operable breast cancer. Cochrane Database Syst Rev Apr 18 (2): CD005002, 2007.

Medioni et al : Neoadjuvant dose-dense gemcitabine plus docetaxel and vinorelbine plus epirubicin for operable breast cancer: Improved prognosis in triple-negative tumors. Drugs R.D. 11: 147-57, 2011.

Padhani AR, et al Diffusion weighted (DW) and dynamis contrast enhanced (DCE) magnetic resonance imaging (MRI) for monitoring anticancer therapy, Targ Oncol 5:39-52 2010

Strieth et al : Paclitaxel encapsulated in cationic liposomes increases tumor microvessel leakiness and improves therapeutic efficacy in combination with Cisplatin. Clin Cancer Res 14: 4603-11, 2008.

Strieth et al : Neovascular targeting chemotherapy: encapsulation of paclitaxel in cationic liposomes impairs functional tumor microvasculature. Int J Cancer 110: 117-24, 2004.

Schmitt-Sody et al : Neovascular targeting therapy: paclitaxel encapsulated in cationic liposomes improves antitumoral efficacy. Clin Cancer Res 9: 2335-41, 2003.

Tofts et al, 1999, Estimating kinetic parameters from dynamic contrast-enhanced MRI of a diffusible tracer: standardized quantities and symbols J Magn Reson Imaging 10:223-232

von Minckwitz G, et al Integrated meta-analysis on 6402 patients with early breast cancer receiving neoadjuvant anthracycline-taxane +/- trastuzumab containing chemotherapy. SABCS 2008 abstr 79

2.0 STUDY DESIGN

2.1 OVERALL STUDY DESIGN AND PLAN

This is a prospective, single-center, open-label phase II trial to explore the therapeutic efficacy of neoadjuvant EndoTAG™-1 in combination with standard paclitaxel followed by a standard FEC regimen in patients with chemotherapy-naïve Her2-negative breast cancer.

Chemotherapy-naïve patients with histologically confirmed BC
available for neoadjuvant chemotherapy

↓

Pre-treatment biopsy: histology and ER/PgR/Her2 status evaluation

↓

If Her2-negative: Patient is eligible

↓

EndoTAG™-1 22 mg/m^2
plus
Paclitaxel 70 mg/m^2

12 weekly infusions

↓

% reduction in MRI-estimated tumour
volume and perfusion

↓

Therapy with FEC regimen:
Fluorouracil 500 mg/m^2
Epirubicin 100 mg/m^2
Cyclophoasphamide 500 mg/m^2

q 3wks x 3 cycles

↓

Surgery
pCR, RCB

Patients with chemotherapy-naïve, resectable breast cancer or breast cancer available for neoadjuvant chemotherapy will undergo baseline evaluation after having signed the informed consent form. Patients meeting all eligibility criteria for participation in this study will be administered:

Weekly treatment with EndoTAG™-1 (22 mg/m^2) plus paclitaxel (70 mg/m^2) for 12 weeks (ET+P) followed by subsequent treatment with the standard FEC regimen (Fluorouracil 500 mg/m^2, Epirubicin 100 mg/m^2, Cyclophosphamide 500 mg/m^2) once every 3 weeks for 3 cycles of therapy.

After receiving neoadjuvant polychemotherapy, patients will be subject to surgery. Following surgery, adjuvant chemotherapy is at the discretion of the investigator; however adjuvant treatment is not part of this protocol.

2.2 TRIAL PERIODS

Screening Visit: Days -14 to -1

Chemotherapy-naïve patients diagnosed for early breast cancer with histologically confirmed Her2-negative tumor status may be screened for participation in the present study. Prior to all screening procedures the investigator is responsible for explaining all aspects of this trial to the patient including potential side effects and alternative treatments available. The investigator must answer all questions by the patient according to his/her best knowledge and must give the patient sufficient time to consider her participation. If the patient wants to participate in this trial, she must first give written informed consent. Only then may the investigator proceed with the screening procedure.

During screening all eligibility criteria will be assessed and evaluations will be performed according to section 5.1 of this protocol.

Enrollment: Day 0

If all inclusion criteria and none of the exclusion criteria are fulfilled, the patient will be enrolled to one the tral. Study treatment has to start within 7 working days after the date of randomization.

Blinding will not be performed.

Treatment Phase:

The treatment phase is displayed in the scheme below. Treatment starts with 12 weekly infusions of taxanes (ET+P) during Visits 1 to 12. During Visit 13, 3 weeks after the final taxane administration, patients will be evaluated for early response of the tumor. At the same visit, administration of the FEC regimen will be started and given once every 3 weeks for 3 cycles.

Surgery:

Surgery will be performed within 28 days to Visit 15 (last administration of FEC regimen).
Pathological complete response (pCR) will be determined by microscopic examination of the excised tissues from the breast and regional lymph nodes.

Follow-up Visit:

A follow-up visit will be scheduled 3 weeks after surgery to collect data on the patients' overall condition. Adjuvant therapy is at the discretion of the investigator and will not be part of this study.

Expected duration of a patients study participation

For each individual patient, study participation will be about 6 months from screening to surgery: 1 to 2 weeks of screening, 11 weeks of treatment with taxanes, 9 weeks of treatment with FEC, 3 weeks to surgery and 3 week of follow-up after surgery for a total of 27 weeks.

Final analysis

Final analysis of data will be performed after surgery of the last patient finishing treatment.

2.3 STUDY OBJECTIVES

Primary objective:

To investigate the activity of EndoTAGTM-1 + paclitaxel combination therapy in patients with Her2-negative BC candidate for neoadjuvant chemotherapy, as measured by the decrease in MRI-estimated tumour volume at the end of EndoTAGTM-1 + paclitaxel administration vs. baseline.

Secondary objectives:

- Decrease in MRI-estimated tumour perfusion at the end of EndoTAGTM-1 + paclitaxel administration
- Pathological complete response (pCR)
- Rate of residual cancer burden at time of surgery
- Safety and tolerability of the neoadjuvant regimen
- Rate of breast-conserving surgery (BCS)
- Rate of clinical complete and partial responses (CR, PR)
- Rate of lymph node-negative disease after treatment (node-NEG)
- Collect frozen tumor biopsies at baseline and surgery for future translational research on DNA and RNA

2.4 STUDY ENDPOINTS

Primary efficacy endpoint:

The percent reduction in MRI-estimated tumor volume at the end of EndoTAGTM-1 + paclitaxel administration vs. baseline.

Secondary efficacy endpoints:

- The percent reduction in linear tumour size as measured on MRI by the greatest linear diameter(s) of the tumour following RECIST criteria
- **The percent reduction in** MRI-estimated tumour perfusion at the end of EndoTAGTM-1 + paclitaxel administration
- **Pathological complete response** (pCR) is defined as the absence of any residual invasive cancer in the breast and the absence of any metastatic cells in the regional lymph nodes at the time of surgery (University of Texas MD Anderson Cancer Center trial's pCR criteria)
- **Residual cancer burden** (RCB) scores are calculated according to the RCB index proposed by Symmans et al (2007)
- Rate of breast-conserving surgery (BCS)
- Rate of clinical complete and partial responses (CR, PR)
- Rate of lymph node-negative disease after treatment (node-NEG)

Safety endpoints:

- **Adverse Events (AEs)**: Incidence and percentage of patients with treatment emergent AEs
- **Laboratory Values:** Number of clinically significant abnormal laboratory values
- **Physical examination and vital signs:** Number and percentage of clinically significant abnormal values
- **Dose variations:** Percentage of patients having dose reductions, delays or discontinuation of study medication

Translational endpoints:

- Collect frozen tumor biopsies at baseline and surgery for future translational research on DNA and RNA

3.0. STUDY HYPOTHESIS

The study hypothesis is that EndoTAG will improve MRI- estimated volume reduction when added to weekly paclitaxel. The null hypothesis is that combination has no or a negligible effect on volume reduction (defined as lower or equal to a 50% decrease) versus the alternative hypothesis that the combination yields at least a 80% average decrease in MRI- estimated volume at the end of weekly paclitaxel and EndoTAG administration from baseline. A standard deviation estimate of 59% was obtained from Delille et al 2003, a multiregimen neoadjuvant chemotherapy study that included 14 patients and for which the average percent MRI- estimated volume decrease was equal to 60%.

4.0 PATIENT POPULATION / ELIGIBILITY CRITERIA

4.1 INCLUSION CRITERIA

1) newly diagnosed, chemotherapy-naïve, histologically-confirmed breast adenocarcinoma with breast infiltrating carcinoma of histological grade > 1 (either operable, or locally advanced or inflammatory) candidate for neoadjuvant chemotherapy
2) Her2-negative tumor, defined according to immunohistochemistry or cytogenetic technique using fluorescent in-situ hybridization (FISH)
3) Either at least one tumor lesion of size ≥ 2 cm or clinical nodal status positive and non-metastatic
4) ECOG performance status 0 or 1
5) Gender: female
6) Age ≥ 18 yrs
7) Negative pregnancy test (females of childbearing potential)
8) Willingness to perform double-barrier contraception during study participation and for 6 months post-chemotherapy treatment (females of childbearing potential)
9) Normal cardiac function, especially normal left ventricular ejection fraction and normal QT interval during ECG (echocardiography) or MUGA (multiple gated acquisition) scan
10) Signed informed consent

4.2 EXCLUSION CRITERIA

1) Metastatic or relapsed breast cancer
2) History of malignancy other than breast cancer within 5 years prior to enrollment, except skin cancer (i.e. basal or squamous cell carcinoma) treated locally
3) Previous or concurrent treatment for breast cancer with any anti-cancer therapy
4) Serious medical conditions like:
    a) congestive heart failure or unstable angina pectoris, previous history of myocardial infarction within 1 year from study entry, uncontrolled arrhythmias
    b) history of significant neurologic or psychiatric disorder that would prohibit the understanding and giving of informed consent, or would interfere with the clinical and radiological evaluation of the central nervous system during study participation
    c) active uncontrolled inflammatory disease (infectious or autoimmune)
    d) active peptic ulcer or unstable diabetes mellitus
    e) severe pulmonary obstructive or restrictive disease

5) Concurrent treatment with hormonal replacement therapy
6) Major surgery within 3 weeks prior to enrollment
7) Results of laboratory tests (hematology, chemistry) outside specified limit
   - WBC ≤ 3 x 10^9/L
   - ANC ≤ 1.5 x 10^9/L
   - Platelets ≤ 100 x 10^9/L
   - Hb ≤ 9.0 g/dl (≤ 5.6 mmol/l)
   - PTT/TNR > 1.5 x ULN
   - AST or ALT > 2.5 x ULN
   - Alkaline Phosphatase > 2 x ULN
   - Total Bilirubin > 1.5 x ULN
8) Pregnancy or nursing status
9) Known positive HIV testing
10) Known hypersensitivity to any component of the EndoTAG™-1, taxane or FEC formulations
11) Concurrent treatment with other experimental products or participation in another clinical trial with any investigational product within 30 days prior to study entry

5.0 EXAMINATIONS

5.1 PRE-TREATMENT EXAMINATIONS AND TUMOR STAGING

During screening visit, the following pre-treatment examinations will be performed within 14 days to randomization:

- signed informed consent
- demographic data, including birth and ethnic origin
- eligibility criteria
- disease history, including date of diagnosis
- medical and surgical history, including past and concurrent clinically significant diseases
- physical examination, including measurements of the breast tumor
- vital signs, including blood pressure (supine), heart rate, and body temperature (oral, axillary, tympanic or rectal), body weight and height. For measurement of body temperature, the same type of measurement is to be used throughout the study
- ECOG performance status
- blood samples for laboratory analyses:
    - hematology including differential blood count
    - clinical chemistry
    - coagulation parameters
- urine analysis
- pregnancy test, if patient is of childbearing potential (serum or urine)
- electrocardiogram and left ventricular ejection fraction (LVEF) assessment by MUGA scan or echocardiography
- tumor imaging can be perfomed within 28 days prior to randomization: bilateral mammography and breast ultrasound, including measurements of the breast tumor; chest-x-ray, bone scan (x-ray or CT-scan in case of hot spots), liver ultrasound; further imaging examination to be performed if lesions suspected in other areas
- Immunohistochemical or cytogenetic analysis of tumor receptor status: test for estrogen, progesterone and Her2 receptors. Analysis prior to given informed consent may be

documented if patient agrees and analyses were performed within 28 days of randomization)
- any adverse event occurring since signature of informed consent
- concomitant medication, including medication within 6 months to screening date

5.2 EXAMINATIONS DURING NEOADJUVANT CHEMOTHERAPY

During **Visits 1 to 12**, the following examinations will be performed after randomization and prior to treatment:

- Physcial examination, including measurements of breast tumor (visit 1,4,7,10,12 only)
- Vital signs, including blood pressure (supine), heart rate, and body temperature (oral, axillary, tympanic or rectal), and weight
- Blood samples for **immediate** laboratory analyses: hematology including differential blood count
- Blood samples for chemistry (visit 1,4,7,10,12 only)
- Blood samples for coagulation parameters (visit 1 and as clinically indicated)
- Adverse events and serious adverse events occurring since last visit
- Any change in concomitant medication since last visit

If all necessary examinations for Visits 1 to 12 are performed and results do not interfere with dosing, patients receive treatment with taxanes according to their allocated treatment group.

3 weeks after the last taxane administration, at **Visit 13**, the following examinations will be performed beforeFEC administration:

- MRI (MRI could be done between 2-3 weeks after the end of paclitaxel/ EndoTAG™-1 administration and must be done before FEC administration)
- Electrocardiogram and left ventricular ejection fraction (LVEF) assessment by MUGA scan or echocardiography should be done.
- ECOG performance status
- Physcial examination, including measurements of breast tumor
- Vital signs, including blood pressure (supine), heart rate, and body temperature (oral, axillary, tympanic or rectal), and weight
- Blood samples for **immediate** laboratory analyses: hematology including differential blood count, chemistry.
- Adverse events and serious adverse events occurring since last visit
- Any change in concomitant medication since last visit

If all necessary examinations for Visit 13 are performed and results do not interfere with dosing, patients receive FEC polychemotherapy.

During **Visits 14 and 15**, the following examinations will be performed prior to treatment:

- Physcial examination, including measurements of breast tumor
- Vital signs, including blood pressure (supine), heart rate, and body temperature (oral, axillary, tympanic or rectal), and weight
- Blood samples for **immediate** laboratory analyses: hematology including differential blood count, chemistry .

- Adverse events and serious adverse events occurring since last visit
- Any change in concomitant medication since last visit

If all necessary examinations for Visits 14 and 15 are performed and results do not interfere with dosing, patients receive FEC polychemotherapy.

The “End of study” visit, **Visit 16**, will be performed 3 week after surgery. The following examinations will be performed:

- ECOG performance status
- Physcial examination, including measurements of breast tumor
- Vital signs, including blood pressure (supine), heart rate, and body temperature (oral, axillary, tympanic or rectal), and weight
- Blood samples for laboratory analyses: hematology including differential blood count, chemistry
- Electrocardiogram and left ventricular ejection fraction (LVEF) assessment by MUGA scan or echocardiography
- Adverse events and serious adverse events occurring since last visit
- Any change in concomitant medication since last visit

A detailed description of the study flow chart is presented in Table at the end of the protocol

6.0 TREATMENT OF PATIENTS

Patients participating in this study will first be treated with EndoTAG™-1/Paclitaxel combination therapy. Patients will then be treated with a standard FEC triple combination therapy. To determine individual doses, body surface area will be calculated based on actual body weight.

For dose adjustments, please refer to chapter 7.0 of this protocol.

6.1 ENDOTAG™-1/PACLITAXEL

EndoTAG™-1:

EndoTAG™-1 is composed of cationic liposomal membranes embedded with paclitaxel and will be provided by MediGene AG, Planegg / Martinsried, Germany.

EndoTAG-1 is formed from the synthetic cationic lipid dioleoyl-trimethylammonium-propane chloride (DOTAP), the natural neutral phospholipid dioleoyl-phosphatidylcholine (DOPC) and the well known cytostatic agent paclitaxel at the ratio 47/50/3 (mM/mM/mM) giving rise, in an aqueous solution of trehalose (97.9 mg/mL), to liposomal membranes embedded with paclitaxel.

The liposomes have an overall positive charge (cationic liposomes).

The embedded paclitaxel complies with the requirements of the US and European Pharmacopoeias (USP, PharmEU).

Recommended International Nonproprietary Name of drug substance (INN): Paclitaxel
Compendial Name: Paclitaxel
CAS Registry Number: [33069-62-4]
Molecular Weight: 853.9 g/mol
Formula: $C_{47}H_{51}NO_{14}$

The other compounds used in this formulation are:

Compound:	Formula:	Function:
DOTAP-Cl	$C_{42}H_{80}NO_4Cl$	Membrane compound
DOPC	$C_{44}H_{84}NPO_8$	Membrane compound
Trehalose	$C_{12}H_{22}O_{11}$	Bulking substance
Citric Acid	$C_6H_8O_7$	Acidifier

EndoTAG™-1 contains 10% trehalose, which is metabolized to glucose *in vivo*. Thus, around 15 g of trehalose are infused per application. Please be aware of this when treating diabetic patients (IDDM) with EndoTAG™-1.

EndoTAG™-1 will be manufactured by Nova Laboratories Ltd, Martin House, Gloucester Crescent, Wigston, Leicester, LE18 4YL, UK. Quality control will be performed by MediGene AG and in part by certified analytical laboratories. The final product will be released by MediGene AG.

EndoTAG™-1 is packed in 100 mL glass vials (hydrolytic class I) with bromobutyl stoppers fixed by standard color coded aluminum flip off caps.

Dose justification:

EndoTAG™-1 has been investigated in several phase I and II studies up to a dose of 66 mg/m^2 in several indications and resulted in a favorable efficacy with an acceptable safety profile. In combination with paclitaxel, only marginal additive toxicity has been observed in patients with advanced TNBC. The best risk-benefit-ratio for this patient population was obtained at a dose of 22mg/m^2 EndoTAG™-1 in addition to 70 mg/m^2 paclitaxel. Therefore in this study it is planned to test the safety and efficacy of a neoadjuvant combination therapy of 22 mg/m^2 EndoTAG™-1 plus 70 mg/m^2 paclitaxel.

Dispensing, Storage and Accountability of EndoTAG™-1

EndoTAG™-1 is delivered to the hospital pharmacy as a sterile, white powder for infusion packaged in glass vials for single use. The investigational medicinal product has to be stored at 2-8°C, protected from light.

Prior to application, EndoTAG™-1 has to be reconstituted with water for injection under aseptic conditions using a safety workbench for cytostatics or similar safety measures. The pharmacist and the investigator have to follow the detailed information on preparation and administration of EndoTAG™-1 and corresponding documentation provided by Medigene AG in a separate document.

After reconstitution the stability is limited to 24 hours at room temperature (in-use stability).

After randomization and before start of treatment phase it can be considered to apply a central venous catheter to the patients (if not already present) to facilitate administration of study medication and blood sampling.

The investigator or pharmacist must maintain accurate records of the receipt of all investigational medicinal product provided by MediGene AG, including date received, batch number, expiration date, patient number, amount received and disposition (dispensation date, amount of investigational medicinal product dispensed and patient identification number).

The hospital pharmacy has to take care of the disposal of used vials and materials in contact with the study medication in compliance to the local regulations concerning hazardous (cytotoxic) waste.

All remaining unused investigational medicinal product has to be returned to the supplier after study termination or destroyed at the site's pharmacy according to standard operating procedures after the written permission of Medigene AG. Destruction of investigational medicinal product has to be documented by the investigator and/or pharmacist. The respective documentation has to be sent to Medigene AG.

Contraindications:

EndoTAG™-1 is contraindicated in patients with known severe hypersensitivity to paclitaxel or to any of its excipient.

EndoTAG™-1 should not be used in patients with baseline neutrophils < 1,000/mm^3.

There is no experience of the use of EndoTAG™-1 in pregnant women. Like other cytotoxic anticancer drugs, EndoTAG™-1 could cause fetal harm when administered to a pregnant woman

and, therefore, is contraindicated during pregnancy. Women of childbearing potential must be advised to avoid pregnancy while being treated with EndoTAG™-1 and within four weeks after discontinuation of EndoTAG™-1 therapy. Should they become pregnant after all, the concerned investigator shall be informed immediately.

It is not known whether EndoTAG™-1 is excreted in human milk. EndoTAG™-1 is contraindicated in breast feeding women. It is recommended that breast feeding be discontinued when receiving EndoTAG™-1 therapy.

Precautions and Warnings:

Infusion associated reactions may occur within minutes of starting the infusion of EndoTAG™-1. These are characterized by symptoms including dyspnea, flushing, chest pain, hypertension, tachycardia, sweating, shortness of breath, chills, back pain, tightness in chest and throat as well as hypotension. Temporarily stopping the infusion usually resolves these symptoms without further therapy. However, medication to treat these symptoms (e.g. antihistamines, corticosteroids, catecholamines, see below) as well as emergency equipment should be available for immediate use. In most patients treatment can be resumed after symptoms have resolved without recurrence. To minimize the risk of infusion reactions, the infusion has to be started slowly and infusion rate should not exceed 1.5ml/min. The patient should be carefully monitored during infusion.

Emergency Medication for infusion related toxicity:

- H2-receptor antagonist i.v., (e.g. cimetidine 300 mg i.v. or ranitidine 50 mg i.v.)
- H1-antihistaminic i.v., (e.g. clemastine 2mg i.v.)

together with other "state-of-the-art" emergency medications (e.g. corticosteroids, catecholamines) have to be kept handy on site.

EndoTAG™-1 contains the cytotoxic drug paclitaxel and, as with other potentially toxic compounds, caution should be exercised in handling EndoTAG™-1. The use of gloves is recommended. If EndoTAG™-1 solution contacts the skin, please wash skin with soap and water immediately and thoroughly. If EndoTAG™-1 gets in contact with mucous membranes, they should be flushed with water immediately and thoroughly.

Any unused product and all equipment used for the preparation and administration of EndoTAG™-1 or contacting EndoTAG™-1 must be disposed of in accordance with local requirements concerning cytotoxic drug products.

Paclitaxel:

Paclitaxel is used in combination (70 mg/m^2) with EndoTAG^{TM}-1. Paclitaxel holds marketing authorizations for treatment of lung, ovarian, breast and head and neck cancer and advanced forms of Kaposi's sarcoma.

Further information on paclitaxel can be found in the respective SPC.

Paclitaxel will be packed according to the manufacturer's description of the locally available product.

For contraindications, precautions and warnings for paclitaxel please refer to applicable SPC.

Please follow the local procedures for handling of cytotoxic drugs.

The investigational sites will need to obtain the paclitaxel supply by their local pharmacy.

For reconstitution of paclitaxel please follow the instructions according to the applicable SPC

Dosing schedules:

EndoTAG™-1/paclitaxel combination therapy:

EndoTAG™-1 will be administered at a dose of 22 mg/m^2 as an i.v. infusion which should be started slowly and increased to a maximum of 1.5 ml/min (15 min at 0.5 ml/min, 15 min at 1.0 ml/min. and thereafter 1.5 ml/min.).

Afterwards, paclitaxel will be administered at a dose of 70 mg/m^2 as an i.v. infusion according to the manufacturer's instructions. Premedication with 10 mg dexamethason i.v. 30 to 60 minutes prior to the paclitaxel infusion is indicated.
Dosing of the combination therapy will be performed on days 1, 8, 15, 22, 29, 36, 43, 50, 57, 64, 71 and 78.

6.2 FEC REGIMEN

The FEC regimen contains the chemotherapeutics fluorouracil, epirubicin and cyclophosphamide.

Fluorouracil (5'FU) has been used to treat various cancers for about 40 years. The dosages used in this protocol for neoadjuvant treatment of early breast cancer are according to the current standard of care. Further information on 5'FU can be found in the respective SPC. 5'FU will be packed according to the manufacturer's description of the locally available product.

Epirubicin holds marketing authorizations for adjuvant treatment of breast cancer. The dosages used in this protocol for neoadjuvant treatment of early breast cancer are according to the current standard of care. Further information on epirubicin can be found in the respective SPC. Epirubicin will be packed according to the manufacturer's description of the locally available product.

Cyclophosphamide holds marketing authorizations for treatment of breast cancer. The dosages used in this protocol for neoadjuvant treatment of early breast cancer are according to the current standard of care. Further information on cyclophosphamide can be found in the respective SPC. Cyclophosphamide will be packed according to the manufacturer's description of the locally available product.

FEC dosing schedule:

5'-Fluorouracil will be administered by i.v. infusion at a dose of 500 mg/m^2 according to the manufacturer's instructions.

Epirubicin will be administered by i.v. infusion at a dose of 100 mg/m^2 according to the manufacturer's instructions.

Cyclophosphamide will be administered by i.v. infusion at a dose of 500 mg/m^2 according to the manufacturer's instructions.

The first cycle of FEC chemotherapy will start 3 weeks after the patient's last administration of taxane therapy. The FEC combination therapy will be administered once every three weeks for three consecutive cycles, i.e. on days 99, 120 and 141 of the patients' study participation.

7.0 DOSE DELAY, OR WITHDRAWAL

To assess whether a patient can be subject to the next dose of study medication, i.e. treatment with E+P, the following analyses will be performed within a maximum time of 24 hours prior to administration of study medication (preferably performed on the day of treatment):

Treatment with E+P can be administered if

hematology analysis are:

- ANC ≥ 1.0 x 10^9/L
- Platelets ≥ 75 x 10^9/L

No non-hematological toxicity > grade 2 precluding treatment

Based on toxicities experienced by the patient, doses will be delayed or patients will be withdrawn from treatment as outlined below.

For dosing delays and withdrawals of the FEC regimen, please refer to the manufacturers' instructions.

7.1 DOSE DELAY

Doses of EndoTAG™-1/paclitaxel combination will be postponed to the next visit if one of the following toxicities of ≥ grade 3 NCI-CTC is observed:

- ANC < 1.0 x 10^9/L (1000/mm^3)
- Platelets < 75 x 10^9/L (75.000/mm^3)
- Non-hematological toxicity, e.g.
    - AST (GOT) or ALT (GPT) > 5.0 ULN
    - Serum creatinine > 3.0 N
- Any other non-hematological toxicity > grade 2 precluding treatment

Delay of dosing is allowed for up to 4 consecutive weeks. The use of G-CSF or GM-CSF to prevent or accelerate recovery from severe hematotoxicity and the prophylactic use of antibiotics is at the discretion of the investigator.

If omission of dosing is needed for more than 4 consecutive weeks, treatment with taxanes will be stopped. The End of study Visit, Visit 18, will be performed and the patient discontinues study participation.

7.2 TREATMENT WITHDRAWAL

Treatment withdrawal will be mandatory in case of:

a) clinical and/or instrumental evidence of congestive heart failure or of any other severe cardiac disease

b) persisting toxicity despite a maximum of four weeks of delay and/or dose-reductions have been implemented according to the guidelines reported in this chapter.

7.4 WITHDRAWAL FROM STUDY PARTICIPATION

Patients have the right to withdraw from the study at any time for any reason without further disadvantages. The investigator also has the right to withdraw patients from the study if it is in the patient's best interest. If any of the screening assessments shows a clinically relevant abnormal finding, in particular a significantly abnormal laboratory parameter, which is in contrast to the eligibility criteria or interferes with the requirements of the study in the opinion of the investigator, the patient will be classified as a screening failure, will not be randomized and dropped out of the study. Nevertheless, the patient has the chance to be re-screened at a later time point. However, if a patient is dropped out of the study after randomization, she is not allowed to be re-screened and enter the study again.

An excessive rate of withdrawals can render the study non-evaluable; therefore, unnecessary withdrawal of patients should be avoided. However, if a safety concern occurs which is regarded as clinically relevant by the investigator, patients must be excluded. Should a patient decide to withdraw, every effort shall be made to complete and report the observations as thoroughly as possible.

All assessments scheduled for the End of Study Visit have to be performed if a patient discontinues treatment premature (except if she discontinues during the screening period).

The following criteria will lead to premature termination of treatment:

- Withdrawal of consent by patient
- Withdrawal by investigator due to safety or ethical concerns
- Postponement of any administration of study drug for more than four consecutive weeks
- Any major surgery
- Chemotherapy other than that indicated in the study
- Progression of disease under investigation
- Pregnancy of a patient

8.0 BREAST CANCER SURGERY AND POST-OPERATIVE THERAPY

Breast cancer surgery will be performed three weeks after the last FEC administration. Breast-conserving surgery or conservative mastectomy will be performed at the discretion of the participating surgeon.

Concomitant ipsilateral axillary dissection will be performed, if applicable. No exclusive sentinel node biopsy will be allowed in this study because neo-adjuvant therapy could interfere with the level of reliability of this surgical procedure. However, sentinel node biopsy will be allowed if followed by standard axillary dissection.

Treatment of patients after surgery can be mandatory and is at the discretion of the investigator. However, any form of adjuvant therapy is not part of this protocol.

Radiotherapy

It is mandatory to administer radiotherapy in case of breast-conserving surgery. Radiotherapy after mastectomy will be administered at the discretion of the investigator. Radiotherapy will be performed after breast cancer surgery. In case of adjuvant hormonal therapy, concomitant radiotherapy will be allowed.

Hormonal therapy
Adjuvant hormonal therapy is recommended in all patients with potentially endocrine-responsive tumors. Administration of adjuvant hormonal therapy will be at the discretion of the investigator.

9.0 EVALUATIONS AND PROCEDURES

9.1 ASSESSMENT OF EFFICACY

Tumor receptor status evaluation

HER-2 receptor status will be evaluated by fluorescent in-situ hybridization (FISH) with the HER-2 spectrum green and CEP17 spectrum aqua probe by Vysis (Illinois, USA). The tumor will be classified HER-2 amplified if the ratio between the HER-2 gene copy number and the centromere 17 copy number will be ≥ 2. (18).

Estrogen and progesterone receptors (ER and PgR) will be evaluated by IHC. ER and PgR scores will be expressed as percentage of tumor cells with positive staining. Tumors will be defined as ER-negative if < 10% of tumor cells will have positive immunostaining. PgR score will not be used to decide whether the patient is eligible for the study (see eligibility criteria, chapter 4.0).

All tumor receptor status evaluations will be performed at the Jules Bordet Institute in Brussels. ER and PgR status will be confirmed centrally at the Jules Bordet Institute with Novocastra antibodies (clone 6F11 for ER; AB for PgR) using an automated immunostainer (Nexes, Ventana).

Tumor biopsy, tissue samples handling, pCR and RCB:

Tumor core biopsy (no fine needle aspiration, no incisional or excisional biopsy) will be performed at surgery.

At the time of pathology examination, the following scenarios will be possible:

a) persistence of macroscopic invasive breast cancer in the breast and/or in the axillary nodes
b) persistence of microscopic invasive breast cancer in the breast and/or in the axillary nodes
c) pathological complete response (pCR): absence of residual invasive breast carcinoma (macro and microscopic) in the breast and in the axillary nodes. Persistence of in-situ carcinoma will not interfere with the definition of pCR.

The definition of pCR will require the examination of a minimum of ten sections from the original site of the primary tumor and will be guided also by the gross examination. All axillary lymph nodes smaller than 1 cm will be entirely examined through 2 mm sections. In case of lymph nodes of at least 1cm, two sections of the macroscopically most suspicious areas will be examined. Evaluation of pCR will be performed according to the University of Texas MD

Anderson Cancer Center trials's pCR criteria. RCB scores will be calculated according to the RCB index proposed by Symmans et al (2007).

MRI-estimated tumour size and perfusion will be assessed using the following methods:

- size will be measured
    - from volume measurement done on the subtraction image from a series of dynamic contrast-enhanced 3D gradient-echo, non fat saturated sequences, obtained by substracting the pre-contrast series images from the peak enhancement series images. The volume shape selection will be done manually using a computer-aided segmentation routine available on the PACS system (Telemis TMRHE ®).
    - from linear size measurement defined as the sum the greatest diameter of the lesion(s) measured on subtraction images(after multiplanar reformation), using the RECIST criteria
- changes in perfusion will be measured using the data from the same series of dynamic contrast-enhanced 3D gradient-echo, non fat saturated sequences, using changes in Ktrans (transfer constant) parameter obtained by applying to the dynamic data a theoretical pharmacokinetic model as defined by Tofts et al which is considered (Padhani et al) as a good parameter to estmate forward leakage of the contrast agent in the tumour and to be a good approximation of the tissue blood flow per unit volume. It will be computed with a home-made evaluation software.

9.2 ASSESSMENT OF SAFETY

The following parameters will be obtained according to the schedule of chapter 5.0:

- Physical Examination (complete clinical evaluation of all body systems)
- Vital Signs (measurement of blood pressure (supine), heart rate, body temperature (oral, axillary, tympanic or rectal) and the patient's weight will be performed. Assessment of the patient's height will be performed during the baseline visit only.
- Clinical laboratory analyses (see below)
- Adverse events (for recording and reporting of AEs please refer to chapter 15.1)

The following laboratory analyses will be performed according to the schedule of chapter 5.0:

Hematology:	Erythrocytes, leukocytes with differential count, hemoglobin, hematocrit, thrombocytes
Chemistry:	SGOT/AST, SGPT/ALT, γ-GT, alkaline phosphatase, total bilirubin, potassium, sodium, calcium, serum creatinine, urea, glucose, albumin
Coagulation Parameters:	PTT, PT, total Fibrinogen
Urine Analysis	Dipstick analysis for glucose, proteins, erythrocutes,leukocytes. Microscopic analysis will be done in case of cellular abnormality
Pregnancy test:	Assessment of serum or urine for pregnancy of female patients of childbearing potential.

The safety profile will be assessed using the NCI CTC version 4.0 as the standard classification and severity grading scale for adverse events (including updates 4.01, 4.02 and 4.03).

9.3 TRANSLATIONAL Research

We will collect frozen tumor samples at baseline and surgery for future translational research on DNA and RNA

10.0 FOLLOW-UP

1 week after surgery a follow-up visit will be scheduled to document the patient's condition after surgery. Examinations of this "End of study" visit, Visit 18, will be performed according the schedule of chaper 5.0.

11.0 STATISTICS

The required number of patients is based on the following assumptions:
The sample size was based on a one-sided t-test for the average percentage decrease in MRI-estimated volume at the end of weekly paclitaxel and EndoTAG administration from baseline The null hypothesis is that combination has no or a negligible effect on volume reduction (defined as lower or equal to a 50% decrease) versus the alternative hypothesis that the new drug yields at least a 80% average decrease in MRI- estimated volume at the end of EndoTAG administration from baseline. A standard deviation estimate of 59% was obtained from Delille et al 2003, a multiregimen neoadjuvant chemotherapy study that included 14 patients and for which the average percent MRI- estimated volume decrease was equal to 60%. For a one-sided significance level of 0.1 and a power of 82%, at least 20 evaluable HER2-negative BC patients are required.

The efficacy and safety analyses will be performed in the intent-to-treat (ITT) population. The ITT population consists of all randomized patients who had at least one administration of study medication.

The primary analysis will consist of a one-sided t-test at the 0.1 significance level.

The efficacy and safety analyses will be also performed in the per-protocol (PP) population. The PP population consists of all patients in the ITT population treated according to the procedures of the protocol without any protocol violations.

Continuous variables will be summarized using standard summary statistics as appropriate (n, mean, standard deviation, median, minimum, maximum, 25th and 75th percentiles). Summary statistics for categorical variables will include frequency counts and percentages, together with 90% confidence intervals.

Subgroup analyses may be included to evaluate efficacy and safety in patients according to hormone receptor status..

12.0 STUDY REGISTRATION AND LOGISTICS

1) Requirements of the Investigators and Study Centers

Investigators:
The investigators are oncologists, surgeons or specialists in internal medicine with a thorough medical background and at least two years experience in the conduct of clinical trials documented by a Curriculum Vitae (CV) which has to be provided by each participating investigator. All investigators have to have appropriate knowledge of Good Clinical Practices and will be well trained in all study specific issues. The study staff at the site has to be well experienced in the treatment of cancer patients, especially in the application of chemotherapeutical agents. In addition, the staff must include a pharmacist or an adequate person with thorough experience in the preparation of chemotherapeutic agents.

Investigational site:
The site must have proper equipment for preparation and administration of the drugs requested in this protocol and for all examinations required and must have easy access to emergency equipment in case it is needed. The investigational site must include or must have access to a laboratory with current accreditation or adequate certification for the analysis of blood samples in hematology, differential blood count, coagulation parameters and blood biochemistry as well as for the analysis of urine samples. The pharmacy of the investigational site must have ample refrigerated storage room with restricted access for storage of the investigational medicinal product provided in this trial (storage between 2 °C and 8 °C). In addition, the site must have enough adequate storage room with restricted access to keep all study related documentation during the active conduct of the study and for archiving for 10 years after the end of the trial.

2) Administrative Structure

This is an investigator initiated trial. The coordinating investigator of this trial is Prof. Ahmad Awada, MD, Institut Bordet Brussels, Belgium. The distribution of responsibilities between all participating investigators and departments of the study site will be defined in a project plan.

All safety laboratory assessments (hematology, differential blood count, coagulation parameters, blood biochemistry, pregnancy test) will be performed in the local laboratories of the study site.

13.0 ETHICS, AUTHORITIES AND INFORMED CONSENT

The study protocol, the patient information sheet, the informed consent form and any other relevant study documentation will be submitted to the ethics committee of the study site. The study will be started only after approval has been obtained. Progress reports and serious adverse events, life threatening problems or deaths have to be reported to the ethics committee.

The guidelines and ethical principles of the World Medical Association Declaration of Helsinki, the requirements of the Directive 2001/20/EC and implementing guidance documents, Guidelines of ICH GCP (CPMP/ICH/135/95) as well as the requirements of national drug and data protection laws and other applicable regulatory requirements will be followed strictly.

The study will be conducted in accordance with this protocol.

The study protocol and related documentation, information about the participating sites as well as the Investigational Medicinal Product Dossier (IMPD) will be submitted to the site's Competent Authority. Local Authorities will be notified in addition as required by national law.

The study will only be initiated after written approval of the Competent Authority has been obtained. Any substantial amendments to the submitted documentation or the organization of the study will be submitted to the Competent Authority and only be implemented upon approval.

The investigator is responsible for ensuring that no patient is subject to any study-related examination or activity before that patient has given informed consent. Written informed consent must be given by the patient after the receipt of detailed information. The verbal explanation will cover all the elements specified in the written information provided for the patient.

The investigator will inform the patient of all aspects pertaining to the patient's participation in the study, such as the aims, methods, anticipated benefits, and potential hazards of the study including any discomfort it may entail. The patient must be given every opportunity to clarify any points she does not understand and if necessary, ask for more information. At the end of the interview, the patient will be given time to reflect. Patients will be required to sign and personally date the informed consent form. They will be given a copy of the informed consent to keep. After completion, informed consent forms will be kept and archived by the investigator in the Investigator's Study File.

It should be emphasized that the patient is at liberty to withdraw her consent to participate at any time, without penalty or loss of benefits to which the patient is otherwise entitled. Patients who refuse to give, or withdraw, written informed consent are not included or allowed to continue to participate in this study.

14.0 DOCUMENTATION

For all patients who give written informed consent, regardless of whether they receive any investigational medicinal product, the investigator must record patient identification data in the "Patient Identification List" (full name, initials, date of birth, patient randomization number). The patient identification list must allow for the definite identification of any patient who takes part in the study. The investigator must keep the patient identification list within the Investigator’s Study File.

A statement acknowledging the participation of a patient in this clinical trial must be documented in the patient's medical file/notes and, where the study physician is not the primary care physician, it is recommended that the patient’s primary care physician is informed of his/her patient’s participation in this clinical study, provided the patient gave her consent.

At the beginning of the study, an Investigator’s Study File will be established at the study site. The investigator/institution is responsible for maintaining the trial documents as specified in the Guideline for ICH GCP (CPMP/ICH/135/95) and as required by the applicable regulatory requirement(s). The investigator/institution must take measures to prevent accidental or premature destruction of these documents.

To protect the patient's identity, each patient will receive a unique patient randomization number, which will be used in lieu of the patient's name when the investigator reports AEs and/or other trial related data. Thus, this number, rather than the patient’s name, will appear on all documents

and will be cross-referenced by the patient's initials.

14.1 ADVERSE EVENT RECORDING AND REPORTING

Recording of Adverse Events (AEs):

An AE is any unintended or unfavorable sign, symptom or disease occurring in the patient after she has signed the informed consent, until 4 weeks after the last investigational medicinal product administration, whether or not the event is believed to be causally related to study medication (investigational medicinal product). In the further treatment phase (FEC regimen and surgery) until the end of study visit such signs only have to be recorded and reported as adverse events if the event is believed to be causally related to the study medication. The definition of an adverse event includes any worsening of conditions that were present at the time of entry into the study (signing of the informed consent). An AE can therefore be any unfavorable and unintended sign (including an abnormal finding), symptom, or disease temporally associated with a medicinal (investigational) product, whether or not related to the medicinal (investigational) product (cf. ICH-E2A).

Laboratory results, which are out of range at the screening assessment (up to 14 days prior to randomization), do not fulfill the definition of an AE since these results represent the baseline information.

Progression of the disease under investigation will not be regarded as adverse event during the course of this trial.

Definition of Serious and Non-Serious Adverse Events

A serious adverse event (SAE) is an adverse event which falls into any of the following categories:

- Results in death
- Is life-threatening
- Requires in-patient hospitalization or prolongation of existing hospitalization
- Results in persistent or significant disability or incapacity
- Is an important medical event
- Congenital anomaly/birth defect

A non-serious adverse event (nsAE) is thus an AE that does not fall into any of the above categories.

When deciding whether an AE meets any of the above listed categories, consideration should be given to the following notes:

A ‘life-threatening’ event is one where the patient was at risk of death at the time of the event. The definition does not refer to an event which hypothetically might have caused death if it were more severe.

Hospital admission is usually interpreted as requiring at least one overnight stay. A requirement for out-patient treatment in an emergency room is not, by itself, an SAE although the event

requiring treatment may be. Elective surgery or other elective procedures which require hospitalization are not SAEs if the condition being treated or investigated was pre-existing at the time of entry into the study and did not worsen during the study. However, any untoward outcome of any such procedure should be reported as nsAE or SAE, as appropriate.

Persistent or significant disability or incapacity means that symptoms which disable the patient, defined as a substantial disruption in a person's ability to conduct normal life functions, do not resolve when study drug is discontinued.

An important medical event is an event that may not be immediately life-threatening or result in death or hospitalization but may jeopardize the patient or may require intervention to prevent one of the outcomes listed in the definition above.

Scientific and medical judgment should be used when deciding whether an event is serious. However, in situations where there is any uncertainty or ambiguity, the event should be managed and reported as if it were serious.

Relation to Investigational Medicinal Product(s)

The relation between an adverse event and the investigational medicinal product(s) will be determined by the investigator on the basis of his or her clinical judgment and the following definitions:

Related: There is a reasonable possibility that the adverse event may have been caused by the investigational medicinal product(s). This definition applies to those adverse events that are considered probably, possibly and unlikely related to the use of the investigational medicinal product(s):

a. **Probable:** An adverse event that follows a reasonable temporal sequence from administration of the investigational medicinal product(s); follows a known response pattern to the investigational medicinal product(s); and, when appropriate to the protocol, is confirmed by improvement after dechallenge; and cannot be reasonably explained by the known characteristics of the patient's clinical state or by other therapies.

b. **Possible:** An adverse event that follows a reasonable temporal sequence from administration of investigational medicinal product(s) and follows a known response pattern to the investigational medicinal product(s) but could have been produced by the patient's clinical state or by other therapies.

c. **Unlikely:** An adverse event for which the reasonable temporal sequence from administration of the investigational medicinal product(s) and the response pattern to the investigational medicinal product(s) is doubtful and which, most probably, have been produced by the patient's clinical state or by other therapies, but a causal relationship of the investigational medicinal product(s) could not be ruled out completely.

Not related: An adverse event for which sufficient information exists to indicate that the etiology is unrelated to the investigational medicinal product(s). At least two of the following variables apply:

1. The adverse event does not follow a reasonable temporal sequence after administration

of the investigational medicinal product(s).

2. The adverse event is readily explained by the patient's clinical state or other therapies.
3. Negative dechallenge - the adverse event does not abate upon dose reduction or cessation of therapy (assuming that it is reasonable to expect abatement of the adverse event within the observed interval).

In case the investigator is unable to decide whether the adverse event may have been caused by the investigational medicinal product(s) or not, the adverse event will be treated as related (following worst case scenario).

Assessment of Intensity of Adverse Events
The intensity (severity) of each AE will be assessed according to the NCI CTC Manual, Version 4.0, May 29, 2009. The following definitions should be used for toxicities that are not defined in the NCI CTC scale:

- **Mild**
  The patient is aware of signs or symptoms, but they are easily tolerated and do not interfere with usual activity.
- **Moderate**
  The signs and symptoms are sufficient to restrict, but not prevent usual activity.
- **Severe**
  The patient is significantly impaired or unable to perform usual activity.
- **Life-threatening**
  The patient was at risk of death at the time of the event. The definition does not refer to an event which hypothetically might have caused death if it was more severe.
- **Death**
  The patient died.

Reporting of Adverse Events (AEs)

Serious adverse events (SAE) during neoadjuvant therapy have to be documented in the CRF. In addition, the Investigator shall promptly notify MediGene within 24 hours of the SAE using the SAE form provided by MediGene, including any follow-up information. All AEs should be followed up until they have resolved or a stable status has been reached.

Information shall be sent via fax to:

MediGene AG

Dr. Heinz Weidenthaler, QPPV

Lochhamer Str. 11

82152 Planegg/Martinsried

Email: drugsafety@medigene.com

Tel. +49 89 8565-3323 (during office hours)

Cell +49 172 8656353 (24-hours emergency phone)

Fax +49 89 8565-3333

The Institution and Investigator shall provide promptly all additional information MediGene reasonably requests or that are necessary to fulfil the reporting requirements under applicable laws and regulations.

MediGene shall generate a SAE report and include such report into its internal Safety Database. The Investigator shall generate and file such documents necessary under applicable laws and regulations. Institution and Investigator are solely responsible to fulfil the Sponsor's requirements in respect to SAEs.

SUSAR

Notification

In case Institution or Investigator becomes aware of a SAE that fulfils the criteria of a Suspected Unexpected Serious Adverse Reaction (as defined in EU Directive 2001/20 EU), ("SUSAR") Institution shall promptly notify and shall cause Investigator to promptly notify MediGene within 24 hours of the SAE including using the SAE form provided by MediGene.

The same contact details and reporting timelines as for other SAEs as outlined in section I. (SAE) are applicable.

Institution and Investigator shall provide promptly all additional information MediGene reasonably requests or that are necessary to fulfil the reporting requirements under applicable laws and regulations.

Filing and expedited reporting

Within two (2) business days, MediGene shall generate a SUSAR report in the CIOMS Format and include such report into its internal Safety Database. Institution shall within seven (7) calendar days after getting knowledge of the SUSAR inform the relevant governmental authorities and the ethics committee using the SUSAR report provided by MediGene. Institution shall provide MediGene with copies of the communication with the authorities and ethics committee(s). Institution and Investigator shall generate and file such documents necessary under applicable laws and regulations. Institution and Investigator are solely responsible to fulfil the Sponsor's requirements with respect to SUSAR's.

All nsAEs and SAEs should be documented in the CRF as specified in this protocol. The

investigator should provide details of the event, as instructed, and classify the event as to its intensity and perceived causal relationship to study medication (investigational medicinal product). Where possible, the event should be described in terms of a diagnosis or syndrome rather than as signs or symptoms. For SAEs, signs and symptoms may form part of the event description.

Consideration should be given to the number of reports which are necessary. Repeated events which are closely linked both in type and time may be described in a single report. Similarly, events which form a well-recognized ‘cascade’ may be described in a single report. However, events which are clearly distinct must be reported separately.

14.2 CASE REPORT FORMS

Specific CRFs will be available for this study and will have to be regularly filled in during study conduct. CRFs and all original data should be readily available for review during scheduled monitoring visits. Any data to be recorded directly on the CRF will be considered to be the source data.

Copies of all pertinent information will be retained by the investigator for a period of at least 15 years from study completion.

14.3 DATA MANAGEMENT

Data will be centralized at the Jules Bordet Institute in Brussels, where data management will be performed

15.0 PUBLICATION POLICY

It is anticipated that the results of this study will be presented at scientific meetings and/or published in a peer reviewed scientific or medical journal. Jules Bordet Institute will provide any prepared abstract or manuscript to Medigene AG for review at least 30 days prior to submission to a publisher for MediGene’s review and comment, to ascertain whether any patentable subject matter or MediGene Confidential Information (other than the results of the clinical trial generated) are disclosed therein.

16.0 APPENDICES

Appendix 1: List of abbreviations

AE	Adverse Event
ALT	Alanine transaminase
ANC	Absolute neutrophil count
ATC	Anatomic Therapeutic Classification
AST	Aspartase transaminase
BW	Body Weight
CMO	Contract manufacturing organization
CR	Complete response
CRF	Case Report Form
CT	Computerized Tomography
CTC	Common Toxicity Criteria
DOTAP	1,2-Dioleoyl-sn-Glycero-3-Phosphocholine
ECOG	Eastern Cooperative Oncology Group
EndoTAG™-1	Lipid-complexed paclitaxel developed on basis of the cationic EndoTAG™ technology (registered trademark in Germany)
FU	Follow-Up
GCP	Good Clinical Practice
G-CSF	Granulocyte colony-stimulating factor
GM-CSF	Granulocyte-macrophage colony-stimulating factor
Hb	Hemoglobin
HIV	Human immunodeficiency virus
ICH	International Conference on Harmonization
IEC	Independent Ethics Committee
IMP	Investigational medicinal product
IR	Incomplete response
i.v.	intravenous
EoT	End of Treatment
IRB	Institutional Review Board
ITT	Intention-to-Treat
LPO	Last patient out
LVEF	Left ventricular ejection fraction
MRI	Magnetic Resonance Imaging
NCI	National Cancer Institute, USA
nsAE	Non-Serious Adverse Event
OR	Objective response
OS	Overall survival
PD	Progressive disease
PK	Pharmacokinetics
PP	Per Protocol
PR	Partial response
PT	Prothrombin time
PTT	Partial Thromboplastin Time
RECIST	Response Evaluation Criteria In Solid Tumors - Guidelines by

	EORTC/NCI working party (Therasse et al. 2000, *Eisenhauer et al.* 2009)
SAE	Serious Adverse Event
SAP	Statistical Analysis Plan
SD	Stable disease
SGOT	Serum glutamic oxalo-acetic transaminase
SGPT	Serum glutamic pyruvic transaminase
SOP	Standard Operating Procedures
SPC	Summary of product characteristics
ULN	Upper limit of normal
WBC	White Blood Cells

Appendix 2

INSTRUCTIONS: After freezing in liquid nitrogen, place the sampling tube with this form in the zip-lock bag and transfer everything into a –80°C freezer (see Biospy sampling and freezing procedure booklet).

NeoEndoTAG-1 trial/ TUMOR SAMPLE FORM

Investigator's Name:________________________ Investigator's No: I__I__I

Investigator's Institution:____

Investigator's country:____

Name of person completing this form: ______________ Signature:_________

Date sample is taken: I__I__I__I__I__I__I__I__I

Patient Initials **Date of Birth** **Patient Hospital Chart No**

I___I___I___I I__I__I__I__I__I__I__I__I I__I__I__I__I__I__I__I__I

Table Study Flow chart

Prior to randomisation		Neo-adjuvant treatment												FEC			3 weeks after Surgery
Weeks	Within 2 weeks	1	2	3	4	5	6	7	8	9	10	11	12	15	18	21	27
Visits	Scr	1	2	3	4	5	6	7	8	9	10	11	12	13	14	15	16
Informed consent	X																
Eligibility criteria	X																
Physical examination	X	X			X			X			X		X	X	X	X	X
Menstrual status/Pregnancy test*	X																
Medical history	X																
Vital signs	X	X	X	X	X	X	X	X	X	X	X	X	X	X	X	X	X
Performance status	X	X			X			X			X			X	X	X	X
Her2 status**	X																
ER/PgR expression**	X																
Breast palpation, tumour measurement & nodal status	X	X			X			X			X			X	X	X	X
Prior/Concomitant medication within 6 month to screening date	X	X			X			X			X			X	X	X	X
Prior to randomisation		Neo-adjuvant treatment												FEC			3 weeks after

																	Surgery
Weeks	Within 2 weeks	1	2	3	4	5	6	7	8	9	10	11	12	15	18	21	27
Visits	Scr	1	2	3	4	5	6	7	8	9	10	11	12	13	14	15	16
Bilateral Mammography	X																
Breast echography including measurement of the breast tumor	X																
Chest X-ray or CT scan and Bone scan	X																
Liver ultrasound	X																
Haematology	X	X	X	X	X	X	X	X	X	X	X	X	X	X	X	X	X
Blood chemistry	X	X			X			X			X		X	X	X	X	X
Coagulation	X																
Urine analysis	X																
ECG and LVEF by MUGA scan or echography	X													X			X
AE and SAE	X																
MRI	X													X			
Residual Frozen Tissue	X																X

*Only for women of childbearing potential
**Her2, ER/PgR expression and tumor imaging within 28 days prior to randomization. Mammography befor surgery can be done between W21 and W24.
